# Inhibitory Mechanism of Combined Hydroxychavicol With Epigallocatechin-3-Gallate Against Glioma Cancer Cell Lines: A Transcriptomic Analysis

**DOI:** 10.3389/fphar.2022.844199

**Published:** 2022-03-22

**Authors:** Amirah Abdul Rahman, Wan Zurinah Wan Ngah, Rahman Jamal, Suzana Makpol, Roslan Harun, Norfilza Mokhtar

**Affiliations:** ^1^ Department of Biochemistry and Molecular Medicine, Faculty of Medicine, Kampus Sungai Buloh, Universiti Teknologi MARA, Cawangan Selangor, Sungai Buloh, Malaysia; ^2^ UKM Medical Centre, UKM Medical Molecular Biology Institute (UMBI), Kuala Lumpur, Malaysia; ^3^ Department of Biochemistry, Faculty of Medicine, UKM Medical Centre, Universiti Kebangsaan Malaysia, Kuala Lumpur, Malaysia; ^4^ KPJ Ampang Specialist Hospital, Ampang, Malaysia; ^5^ Department of Physiology, Faculty of Medicine, UKM Medical Centre, Universiti Kebangsaan Malaysia, Kuala Lumpur, Malaysia

**Keywords:** glioma, epigallocatechin-3-gallate, hydroxychavicol, synergism, gene expression, apoptosis, transcriptomic

## Abstract

Emerging reports have shown therapeutic potential of hydroxychavicol (HC) and epigallocatechin-3-gallate (EGCG) against cancer cells, however high concentrations are required to achieve the anticancer activity. We reported the synergy of low combination doses of EGCG+HC in glioma cell lines 1321N1, SW1783, and LN18 by assessing the effects of EGCG+HC through functional assays. Using high throughput RNA sequencing, the molecular mechanisms of EGCG+HC against glioma cell lines were revealed. EGCG/HC alone inhibited the proliferation of glioma cell lines, with IC50 values ranging from 82 to 302 µg/ml and 75 to 119 µg/ml, respectively. Sub-effective concentrations of combined EGCG+HC enhanced the suppression of glioma cell growth, with SW1783 showing strong synergism with a combination index (CI) of 0.55 and LN18 showing a CI of 0.51. A moderate synergistic interaction of EGCG+HC was detected in 1321N1 cells, with a CI value of 0.88. Exposure of 1321N1, SW1783, and LN18 cells to EGCG+HC for 24 h induces cell death, with caspase-3 activation rates of 52%, 57%, and 9.4%, respectively. However, the dose for SW1783 is cytotoxic to normal cells, thus this dose was excluded from other tests. EGCG+HC induced cell cycle arrest at S phase and reduced 1321N1 and LN18 cell migration and invasion. Combined EGCG+HC amplified its anticancer effect by downregulating the axon guidance process and metabolic pathways, while simultaneously interfering with endoplasmic reticulum unfolded protein response pathway. Furthermore, EGCG+HC exerted its apoptotic effect through the alteration of mitochondrial genes such as MT-CO3 and MT-RNR2 in 1321N1 and LN18 cells respectively. EGCG+HC dynamically altered DYNLL1 alternative splicing expression in 1321N1 and DLD splicing expression in LN18 cell lines. Our work indicated the pleiotropic effects of EGCG+HC treatment, as well as particular target genes that might be investigated for future glioma cancer therapeutic development.

## 1 Introduction

Gliomas are the most frequent primary intracranial tumour, accounting for 81% of all malignant brain tumours. Although relatively uncommon with an annual incidence of around 5 cases per 100000, the median survival of glioblastoma patients are around 15 months even after rigorous combination treatment of surgery, chemotherapy and radiotherapy, with and the median progression-free survival ranged from 6.2 to 7.5 months (Liang et al., 2020). Human cancer is a complicated disease, thus, alternative methods for cancer management and treatment and a better understanding of the treatments’ mechanism of action are required to improve patients’ quality of life (Pangal et al., 2021).

Dietary bioactives with high effectiveness and little side effects are preferred as alternatives to synthetic therapies, with a variety of negative side effects. At the moment, the search for combination therapy is of interest as this approach may reduce the development of drug resistance, as well as provides opportunity to discover potential cancer medicines (Maruca et al., 2019). Polyphenols are one of the major classes of phytochemical that is well-known for its disease-fighting effects.

The health benefits, the relatively low side effects and the origin from natural sources may provide added benefits and have resulted in continued interest for these bioactives. They are thought to play two roles: one that modulates chemopreventive benefits by improving antioxidant defences and the ability to scavenge ROS, hence lowering oxidative stress, and the other that targets chemotherapeutic effectiveness by inducing cellular stress (ROS levels) (Surh, 2011). At low concentrations and in normal cells, phenolic compounds may act as cancer preventive agents (Khurana et al., 2018). Some polyphenols species can act as prooxidants, enhancing its chemotherapeutic action, by generating high levels of ROS and eventually induces DNA damage and apoptosis (Kirtonia et al., 2020).

The cancer-preventive effects of (−)-epigallocatechin-gallate (EGCG) on cells *in vitro*, in animal models and within clinical studies have been previously reported (Peter et al., 2016). The anti-cancer effect of EGCG is proposed to originate from its antioxidant activity, through the induction of phase II enzymes, and manipulation of signal transduction pathways such as JAK/STAT (Xiao-Mei et al., 2016), MAPK, VEGF and PI3K/AKT (Liu et al., 2013). The mechanism of EGCG also includes epigenetic regulatory alteration, altering DNA methyltransferase (DNMT), histone deacetylase (HDAC), and miRNA expression (Zhang et al., 2015; Yamada et al., 2016).

Hydroxychavicol is a less studied phenolic compound derived from *Piper betle* leaf extract. Emerging reports including our own have shown the potent activity of HC in impeding cell proliferation in glioma cells (Abdul Rahman et al., 2014), and inhibiting prostate cancer cell cycle progression (Gundala et al., 2014). The efficacy of HC in inhibiting prostate tumour xenografts and chronic myeloid leukemia (CML) cells is suggested to be attributed to its selective prooxidant activity by reactive oxygen species (ROS) generation and induction of caspase-mediated apoptosis (Gundala et al., 2014) and/or by caspase-independent manner via apoptosis inducing factor (AIF) (Chowdhury et al., 2013) to eliminate cancer cells.

We hypothesize that the combination of these phenolic compounds may enhance the therapeutic activity as they impact on a number of pathways in tumour progression. Although studies have reported that EGCG or HC induce cell death without adversely affecting normal cells (Gundala et al., 2014; Meng et al., 2019), a high concentration of EGCG/HC is usually needed for the treatment to be effective on cancer cells. A high dose of EGCG or HC might result in cytotoxicity in normal cells. Therefore, a combination of low concentrations of EGCG+HC may be more effective in killing cancer cells, compared to a single high concentration treatment, as combination treatments may have pleotropic effects, targeting several pathways.

Therefore, we aim to investigate the synergistic interaction of phytochemicals by examining the effect of EGCG or HC singly and its combination against 1321N1, SW1783 and LN18 cell proliferation, cell cycle progression, migration/invasion and colony formation. The molecular mechanisms of EGCG+HC against glioma cell lines were elucidated using high throughput RNA sequencing. Until now, no direct evidence has shown on the anticancer effect of ECGC+HC in different stages of glioma cells.

## 2 Materials and Methods

### 2.1 Reagents and Chemicals

Hydroxychavicol (HC) was bought from Hangzhou Imaginechem Co. Ltd. (Hangzhou, China) and (−)-epigallocatechin-3-gallate (EGCG) was bought from Sigma-Aldrich (United States). CellTiter 96^®^ AQueous Non-Radioactive Cell Proliferation kit (Promega, United States), FITC Active Caspase-3 and Annexin V-FITC Apoptosis Detection Kit, BD CycleTEST™ PLUS DNA Reagent kit from BD Biosciences (United States) and QCMTM 24-well Cell Invasion/Migration Assay kit (ECM550 and ECM508) (Millipore, United States). All of the other chemicals utilised were of analytical grade.

### 2.2 Cell Line and Culture Environment

The human glioblastoma cell line 1321N1 (Grade II) was bought from the European Collection of Cell Culture (ECACC), while the American Type Culture Collection (ATCC) supplied SW1783 (Grade III) and LN18 (Grade IV) cell lines (Manassas, United States). 1321N1 and LN18 were cultured in Dulbecco’s modified Eagle medium (DMEM) supplemented with penicillin/streptomycin, 10% or 5% foetal bovine serum (FBS) respectively, in a humidified incubator at 37°C in an atmosphere of 95% air and 5% CO2. SW1783 was grown in Leibovitz, 10% FBS, under a 100% air atmosphere. The medium was replaced three times a week, and the cells were passaged with accutase.

### 2.3 Natural Compound Treatments

Fresh EGCG stock solutions were made in culture growth media, whereas HC stock solutions were prepared in 100% ethanol and kept at −20°C. Vehicle control was added with 0.1% ethanol.

### 2.4 Cell Viability Determination

Glioma cancer cell viability treated with combined EGCG+HC or EGCG/HC singly was determined using the Cell Proliferation Assay (Promega, United States), as previously described [11]. Cells were seeded at 1×10^4^ cells per well in 96-well plates. After a 24-h incubation period, the media was withdrawn and 100 μl of medium were added, which contains a range of concentration for EGCG (50, 100, 150, 200, 300 μg/ml) or HC (50, 100, 150, 200 μg/ml). EGCG+HC compounds were titrated to a range of concentrations (1, 10, 50, 100 μg/ml). The treatments were incubated for 24 h. The media was then carefully removed, replaced with new medium, and 20 μl of [3-(4,5-dimethylthiazol-2-yl)-5-(3-carboxymethoxy-phenyl)-2-(4-sulfophenyl)-2-(4-sulfophenyl)-2H-tetrazolium, inner salt] (MTS) was added to each well and incubated for 2 h at 37°C. In a VersaMax ELISA microplate reader, absorbance was measured at 490 nm (Molecular Device, United States). At each concentration, the percentage of viable cells was estimated by dividing the absorbance (490 nm) of treated cells by that of control cells. The cell viability (%) versus concentrations graph was used to calculate the half maximum inhibitory concentration (IC50). All tests were carried out in three independent experiments.

### 2.5 Apoptosis Assay Using Active Caspase-3 and Annexin V-Propidium Iodide Staining

Cells were seeded at a density of 5 × 10^5^ cells/dish in a 60 mm culture dish for all functional assays. EGCG was dissolved in culture medium, while HC was dissolved in ethanol and added to the culture media with stipulated concentration. Cells were collected after 24 h and washed twice with PBS. The assays were carried out as specified in the manufacturer’s procedure. In brief, for active caspase-3 assay, cells were fixed in BD Cytofix/Cytoperm solution, incubated on ice for 20 min, washed with BD Perm/Wash buffer, and then incubated for 30 min at 25°C with FITC rabbit anti-active caspase-3 antibody. For annexin V-propidium iodide staining, cells were resuspended in 1X binding buffer. Annexin-V FITC and propidium iodide (PI) were added and incubated in the dark for 15 min at 25°C. The BD FACSCanto™ flow cytometer and CellQuest Pro (IVD) software (Becton Dickenson, United States) were used to detect fluorescence from a population of 1 × 10^5^ cells. Three independent tests were carried out in triplicate.

### 2.6 Analysis of Cell Cycle Progression

The cells were prepared according to the manufacturers’ protocol. Cells were trypsinized, washed, and fixed in Buffer Solution at 4°C. After 10 min of incubation with trypsin buffer at 25°C, 200 μl of trypsin inhibitor and RNase buffer solution were added and incubated for another 10 min. In a dark room, 200 μl of propidium iodide stain solution were added to the mixture and incubated on ice for 10 min. The analysis were done using the BD FACSAria™ flow cytometer, FACScan and ModFit software (Becton Dickenson, United States).

### 2.7 Wound Healing Test

The wound healing test was carried out as previously reported, with several modifications (Liang et al., 2007). After 24 h of plating in a 6-well plate, cells were scratched with a 200 μl sterile pipette tip, rinsed three times with PBS, and incubated with the treatments for another 24 h. The cells were rinsed twice with PBS before being examined and photographed using a Nikon Eclipse TS100 phase-contrast microscope. Using the NIS-Elements imaging programme, the migration percentage was estimated by comparing cells which migrated into scratched regions to 0 h cells.

### 2.8 Transwell Cell Invasion and Migration Assay

The assays were prepared according to the manufacturers’ protocol (Millipore, United States). Both invasion and migration kits utilized an 8 μm pore size polycarbonated membrane insert. A thin layer of ECMatrix™ were pre-coated on the insert for invasion kit which function to seal the membrane pores and block the non-invasive cell migration. Briefly, 300 μl of cells re-suspended in a serum-free medium was added to the upper chamber, while the bottom well was filled with 500 μl of complete culture medium or treatment. Following 24 h incubation, unmigrated cells were removed from the upper chamber, and the migration insert containing migrated cells were transferred into a clean well containing 400 μl of staining buffer. After 20 min incubation at 25°C, the inserts were rinsed in water, and unmigrated cells were removed from the inside of the insert with a cotton-tipped swab. After drying, the stained inserts were placed to a clean well containing 200 μl of Extraction Buffer for 15 min at 25°C. 100 μl of the mixture solution was pipetted a 96-well plate and the absorbance was measured at 560 nm.

### 2.9 Colony Formation Assay

Following a 24 h treatment with combined EGCG+HC and EGCG/HC singly, approximately 400 cells were plated in a 21 cm^2^ culture dish. The cells were grown in a complete media for 12 days and the medium were replaced every 3 days. On day-12, the colonies formed were washed with PBS, and then fixed for 30 min with a mixture of crystal violet solution and methanol (1:1). The plate were rinsed three times with distilled water to remove any excess staining. ChemiDocTM MP (Biorad, United States) was used to capture the images of the stained plates, and Cell Counter v0.2.1 (http://nghiaho.com/?page id=1011) was used to count the colonies. Each treatment was carried out in triplicate.

### 2.10 Statistical Evaluation

Isobologram analysis based on the Chou-Talalay technique (Chou and Talalay, 1984; Zhao et al., 2004) was used to determine the interaction between the two treatments, with the output represented as combination indexes (CI). The CI between two compounds A and B is as follows:

$$\mathrm{CI}=\frac{d_{1}}{\left(D_{m}\right)1}+\frac{d_{2}}{\left(D_{m}\right)2}$$

Where; CI: combination index *d*_1_: the IC_50_ of combination dose for compound 1 *d*_2_: the IC_50_ of combination dose for compound 2 (*D*_m_)_1_: the IC_50_ dose for compound 1 (*D*_m_)_2_: the IC_50_ dose for compound 2.

The magnitude of synergism/antagonism were measured using CI values. CI values between 0.9 and 0.85 indicate mild synergy, those between 0.7 and 0.3 indicate strong synergistic interactions between the treatments. A near additive effect is shown by CI values ranging from 0.9 to 1.10. SPSS 16.0 software was used to analyse the two-tailed Student’s t-test for comparison with vehicle control only (cell viability with single treatments), or two-way ANOVA for multiple comparisons of apoptosis, cell cycle, migration/invasion, and colony formation tests where *p* < 0.05 were considered statistically significant. The data were presented as mean ± standard deviation (SD).

### 2.11 RNA Library Preparation and Sequencing

After 24 h of glioma cells treatment, total RNA was using TRI Reagent^®^ (Molecular Research Center, United States). RNase-free DNase treatment was used to remove contaminating DNA (QIAGEN), and RNA was purified using the RNeasy Mini Kit (QIAGEN). The quality of the RNA was then determined using an Agilent Bioanalyzer 2100 (Santa Clara, United States) and all readings have a minimum RIN score of 9.5. The Qubit 2.0 Fluorometer was used to measure the amount of RNA (Life Technologies, United States). The cDNA libraries were prepared according to the protocol outlined in the TruSeq RNA sample Preparation Kit v-2 (Illumina, San Diego, United States). The description of the procedure may be found elsewhere (Abdul Rahman et al., 2019). A total of 10–12 samples per lane were multiplexed and sequenced on an Illumina HiSeq 2500 using the paired-end cluster generation kit (Illumina).

### 2.12 RNA-Sequencing Data Processing and Pathway Analysis

The analysis of RNA-seq data was previously described elsewhere (Abdul Rahman et al., 2019). For quality control, RNA-seq raw data was trimmed at a PHRED score of <Q25, with a read length of at least 33 bp, and read quality was evaluated for each sample using FastQC. A total of 28–47 million paired end reads were aligned and mapped against the Human Genome version 37, GRCh37/hg19. Significant differences in gene/transcript expression were determined for pairwise comparisons between two sets of samples using the Empirical analysis of DGE (Robinson and Smyth, 2008) by Negative Binomial distribution followed by Bonferroni multiple testing correction (MTC) and Benjamini–Hochberg false discovery rate (FDR) using the CLC Genomics Workbench (7.0.6 version), and by Cuffdiff 2.0 in Tuxedo Suite pipeline (Trapnell et al., 2012). For each comparison, the intersection of the significant genes discovered using both algorithms were deemed differentially expressed.

Pathway Studio (Ariadne Genomics, United States) was used for network analysis, which included Gene Significant Enrichment Analysis (GSEA), Fisher Exact Test (FET), and subnetwork analysis of RNA-seq data, using the mean of RPKM values, and the gene expression log ratio of treatment to control cells were calculated. DAVID Bioinformatics Resources 6.7 was also used for gene ontology annotation. In the subnetwork analysis, genes were deemed key regulators of a network if they controlled five or more gene targets. These networks give a global picture of potentially important, interacting partners of genes that have undergone significant alterations. For the assessments of alternative splicing expression, two types of analyses were performed using the Partek^®^ Genomics Suite (Partek Inc., United States) and the Tuxedo Suite (Tophat and Cuffdiff 2.0). Alternative splicing entities expressed from both platforms were overlapped (Supplementary Tables S1–S3). The expression of alternative splicing events in both analyses was then overlapped to the list of transcripts expression at *p ≤* 0.05 with FC ≥ 1.5, to get the list of alternative splicing events with a significant differential transcripts expression (Supplementary Tables S1–S3).

### 3.13 Validation of Gene Expression Data by Quantitative Real-Time PCR (qPCR)

The genes were chosen for validation because they either demonstrated the most significant increase or reduction in response to EGCG+HC therapy, are a central gene, or are important to cancer formation. 300 ng of total RNA were used for cDNA synthesis using the iScript™ cDNA Synthesis kit (BioRad, United States). TaqMan^®^ Gene Expression Assays and TaqMan^®^ Fast Advanced Master Mix (Applied Biosystem, United States) were used for the amplification of genes and transcripts. cDNA synthesis reactions were performed on Veriti^®^ Thermal Cycler (Applied Biosystems) and qPCR were performed on CFX96 Touch™ C1000 Touch Thermal Cycler (BioRad, United States). The data were normalized to the expression of housekeeping genes TATA box binding protein (*TBP*) or glucuronidase, beta (*GUSB*) and analyzed using the standard 2^-∆∆CT^ method.

## 3 Results

### 3.1 Treatment of EGCG or HC Singly and Its Combination Decreases Glioma Cell Viability

**FIGURE 1:**
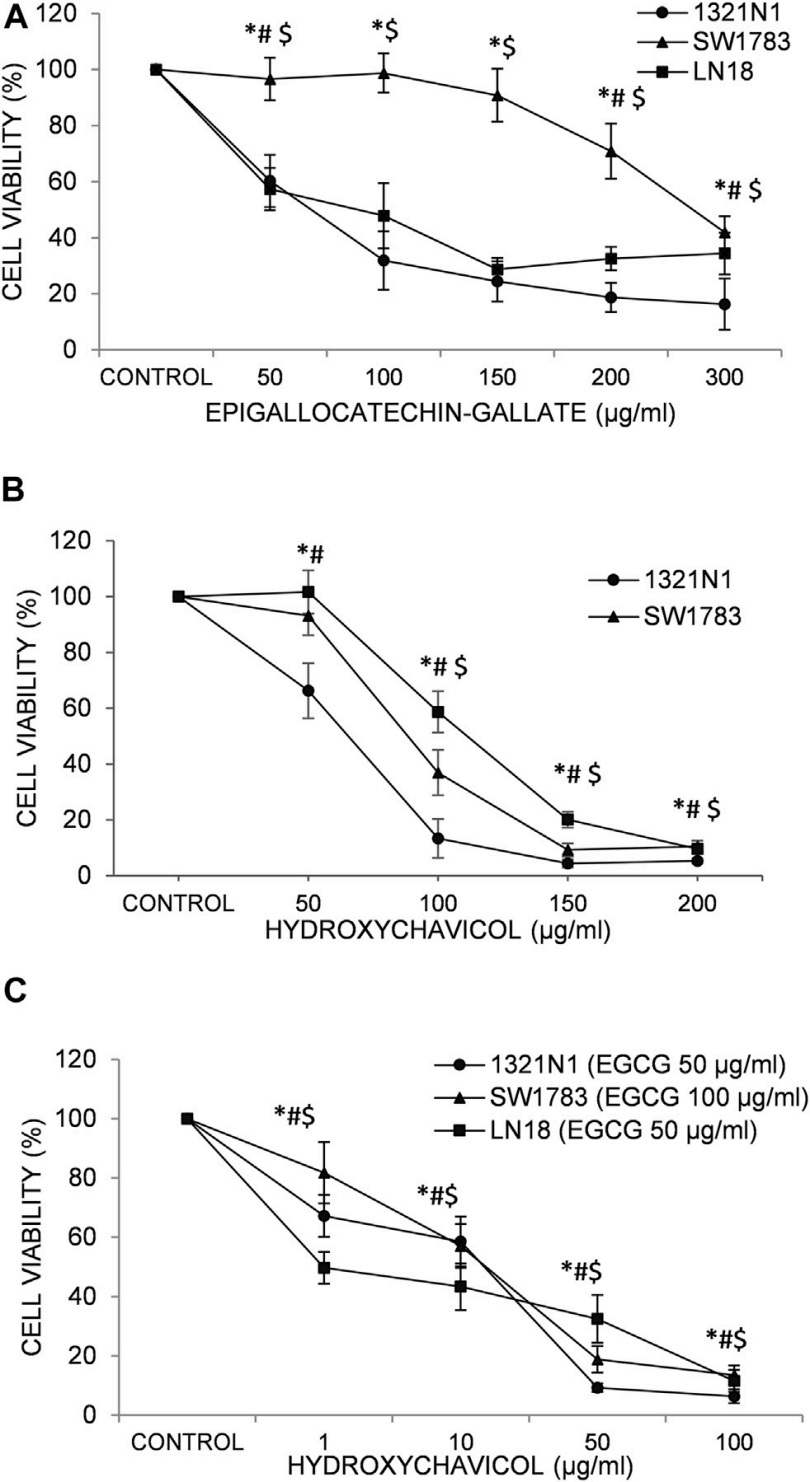
The effects of **(A)** epigallocatechin-3-gallate (EGCG); **(B)** hydroxychavicol (HC); and **(C)** EGCG+HC on 1321N1, SW1783, and LN18 after 24-h. The data reflects the mean±SD of three independent experiments. *p < 0.05 compare to 1321N1vehicle control, #*p* < 0.05 compare to SW1783 vehicle control, and $*p* < 0.05 compare to LN18 vehicle control.

We have previously reported the effect EGCG and HC singly on glioma cell viability (Abdul Rahman et al., 2014;Rahman et al., 2014). The optimum IC50 doses for EGCG, HC and its combination for each cell lines were obtained by performing an initial dose response curve (Figure 1). The growth of 1321N1, SW1783 and LN18 cells was inhibited with the inhibitory concentration at 50% cell death (IC50) values for EGCG ranging from 82 to 302 µg/ml (Rahman et al., 2014) and HC (Abdul Rahman et al., 2014) with values of IC50 between 75–119 µg/ml (Table 1). The cytotoxicity induced by EGCG and HC was found to be dose dependent with 58–84% and 90–95% inhibition respectively achieved after 24 h of treatment (Figure 1).

**TABLE 1:** 50% Inhibitory concentration (IC50) of EGCG and HC on 1321N1, SW1783 and LN18 cells. Viable cells (%) were expressed as the mean ± SD of three independent experiments.

Cell lines	Compound	IC50 value (µg/ml)	Viability (% cells)^a^
Grade II	Epigallocatechin-gallate (EGCG)	82 ± 12.31	16.3 ± 9.2
1321N1	Hydroxychavicol (HC)^b^	75 ± 7.51	5.2 ± 0.89
Grade III	Epigallocatechin-gallate (EGCG)	302 ± 9.10	41.9 ± 5.74
SW1783	Hydroxychavicol (HC)^b^	95 ± 5.83	10.5 ± 2.04
Grade II	Epigallocatechin-gallate (EGCG)	134 ± 11.36	34.4 ± 7.41
LN18	Hydroxychavicol (HC)^b^	119 ± 7.77	9.6 ± 1.66

a Cell viability (%) following a 24 h treatment of the highest concentration value of each compound.

b The results for the treatment of EGCG and HC singly against the viability of glioma cells have been published previously (Abdul Rahman et al., 2014; Rahman et al., 2014).

**FIGURE 2:**
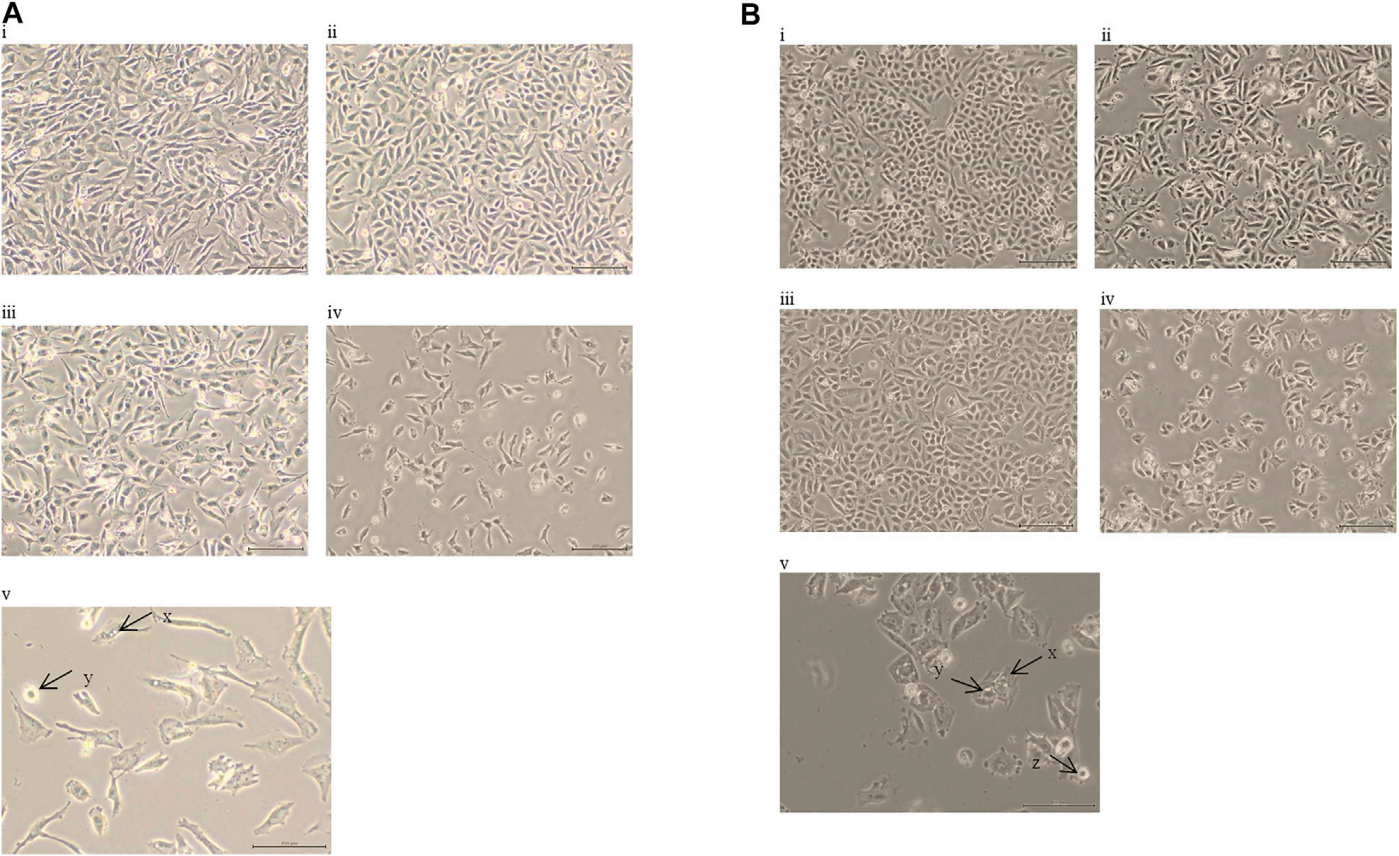
**(A)** Morphological alterations in 1321N1 cells following treatment with i. vehicle control cells, ii. EGCG 50 µg/ml, iii. HC 20 µg/ml, iv. 50 µg/ml of EGCG + 20 µg/ml of HC, v. scale 100 µm; 50 µg/ml of EGCG + 20 µg/ml of HC; for 24 h (40× magnification). The arrows indicate; x. vacuolated cytoplasm and y. cell shrinkage; **(B)** Morphological alterations in LN18 cells following treatment with i. vehicle control cells, ii. EGCG 50 µg/ml, iii. HC 10 µg/ml, iv. 50 µg/ml of EGCG + 10 µg/ml of HC, v. scale 100 µm; 50 µg/ml of EGCG + 10 µg/ml of HC; for 24 h (40× magnification). The arrows indicate; x. vacuolated cytoplasm, y. the collapse of nucleus, and z. cell shrinkage.

The lower IC50 values of EGCG (50/100 μg/ml; compared to the IC50 values of EGCG obtained, ranging from 82 to 302 μg/ml (Table 1)) were titrated on a range of HC concentrations (1–100 μg/ml). Results obtained showed lower IC50 values ranging from 10 to 25 μg/ml for HC when combined with EGCG (Table 2), compared to HC treatment alone on glioma cells (Table 1). Moreover, the combination dose of EGCG and HC were observed to induce morphological changes in the glioma cells by microscopic examination (Figures 2A,B).

**TABLE 2:** The ratio of combined EGCG and HC at 50% inhibitory concentration (IC50) of 1321N1, SW1783, LN18 cells and its combination index (CI).

Type of cell line	EGCG:HC	IC50^a^ [µg/ml]	EGCG^b^ [µg/ml]	HC^b^ [µg/ml]	Combination Index^c^ (CI)
1321N1	5: 2	20	82	75	0.88 ± 0.05
SW1783	4: 1	25	300	95	0.54 + 0.05
LN18	5: 1	10	134	119	0.43 + 0.06

a IC50 of combined compounds.

b IC50 of compound A or B.

c CI < 1.0 indicates synergism; 0.9 < CI < 1.10, near additive; CI > 1.10 indicates antagonism.

### 3.2 Isobologram Analysis of Treatments

The suppression of cell viability was shown to be stronger in the combination of EGCG+HC at lower concentrations than either component alone (Figure 1C, Table 2). An isobologram plot was done for synergism analysis (Supplementary Figure S1) and to determine the combination index (CI). Synergistic interactions were seen in all glioma cells treated with combined sub-effective concentrations of EGCG and HC, as shown in Table 2, with CI = 0.88 for 1321N1, CI = 0.54 for SW1783, and CI = 0.43 for LN18 cells. The combined EGCG+HC treatment for 1321N1 is 50 μg/ml EGCG + 20 μg/ml HC, SW1783 is 100 μg/ml EGCG + 25 μg/ml HC and LN18 is 50 μg/ml EGCG + 10 μg/ml HC.

### 3.3 Combined EGCG+HC Induced Apoptosis by Triggering Caspase-3 Activation

**FIGURE 3:**
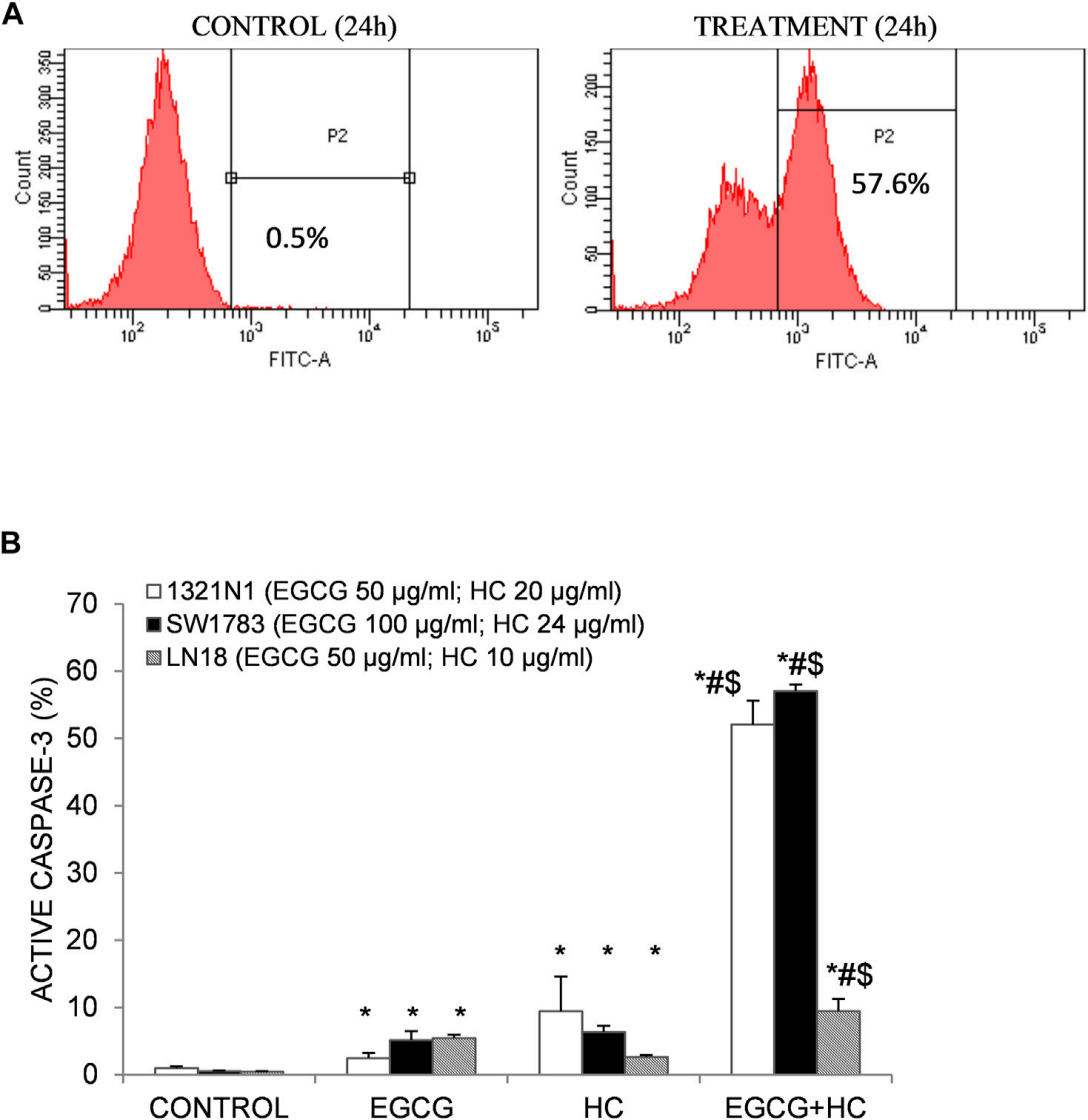
**(A)** Flow cytometry detection of apoptosis following caspase-3 antibody staining. An example of an active caspase-3 detection diagram using 1321N1 cells: viable cells are indicated in the left quadrant, while active caspase-3 is displayed in the right quadrant (P2). **(B)** As evidenced by the presence of active caspase-3, combined EGCG+HC triggered stronger induction of apoptosis in 1321N1, SW1783, and LN18 cells than either EGCG/HC alone. Data represents mean±SD of three independent experiments. *p < 0.05 compare to vehicle control, #*p* < 0.05 compare to EGCG, $*p* < 0.05 compare to HC.

The synergy effect of combined EGCG+HC on 1321N1 and SW1783 amount to multiplication of caspase-3 activation of 52 and 57% respectively than by either EGCG or HC alone (Figure 3). Induction of active caspase-3 in LN18 EGCG+HC treated cells was observed to be the lowest (9.4%) compared to both 1321N1 and SW1783 (Figure 3).

**FIGURE 4:**
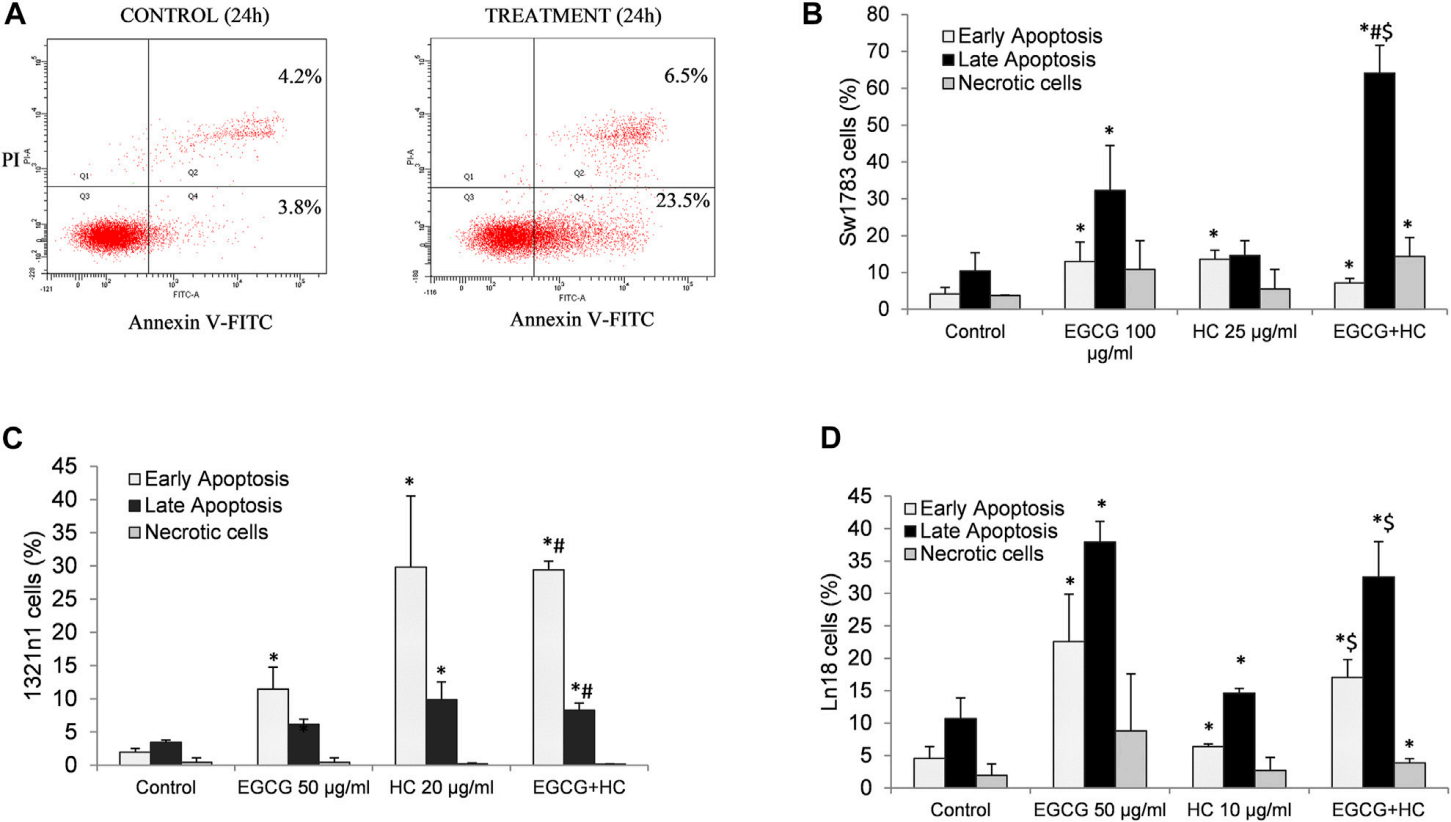
**(A)** Flow cytometry detection of apoptosis following annexin V-FITC/propidium iodide (PI) labelling. The lower left quadrant (Q3) contains viable cells, the lower right quadrant (Q4) contains early apoptotic cells, the upper right quadrant contains late apoptotic cells, and the upper left quadrant contains non-viable necrotic cells (Q1). A bar graph displaying the mean values from three independent experiments for **(B)** 1321N1, **(C)** SW1783, and **(D)** LN18. *p < 0.05 compare to control early or late apoptosis respectively, #*p* < 0.05 compare to EGCG early or late apoptosis respectively, $*p* < 0.05 compare to HC early or late apoptosis respectively.

Figure 4B shows that the percentage of both early (29.4%) and late (8.3%) apoptotic cells for EGCG+HC treatment in 1321N1 cells increased significantly compared to vehicle control and EGCG, but no changes were observed when compared to HC treatment alone. An increase of early (17.1%) and late (32.5%) apoptosis in LN18 treated with combined EGCG+HC was observed when compared to vehicle control and HC alone, but no changes were observed for early apoptosis when compared to EGCG treatment alone (Figure 4D). While for SW1783, a significant increase was seen in the percentage of late apoptosis (64%) for cells treated with EGCG + HC compared to vehicle control, ECGC (32%) or HC (14.6%) treatment alone (Figure 4C).

### 3.4 The Effect of Combined EGCG+HC on Normal Cells

**FIGURE 5:**
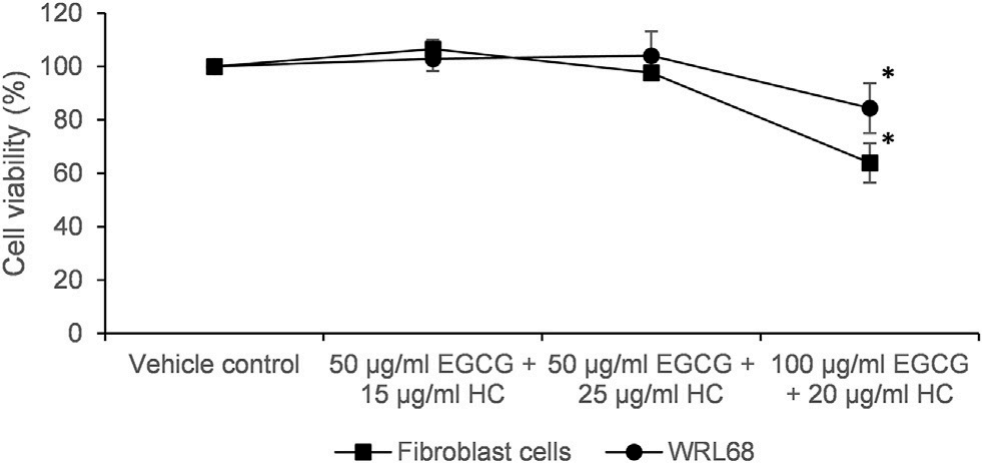
Cytotoxicity of EGCG+HC on normal cells; foreskin fibroblast cells and WRL68 using the IC50 of combined EGCG+HC treatment: 50 μg/ml EGCG + 20 μg/ml HC (dose for 1321N1), 100 μg/ml EGCG + 25 μg/ml HC (dose for SW1783) and 50 μg/ml EGCG + 10 μg/ml HC (dose for LN18).

Figure 5 shows that no cytotoxicity was observed on normal cells (foreskin fibroblasts cells and WRL68) for combined EGCG+HC treatment using IC50 doses of 1321N1 (50 μg/ml EGCG + 20 μg/ml HC) and LN18 (50 μg/ml EGCG + 10 μg/ml HC) obtained from the MTS assay data. However, a significant reduction of cell proliferation was seen on normal cells using the combination dose of SW1783 (100 μg/ml EGCG + 25 μg/ml HC) (Figure 5). For this reason, the dose of 100 μg/ml EGCG + 25 μg/ml HC on SW1783 was excluded for further testing.

### 3.5 The Effect of Combined EGCG+HC on Cell Cycle Progression of Glioma Cells

**FIGURE 6:**
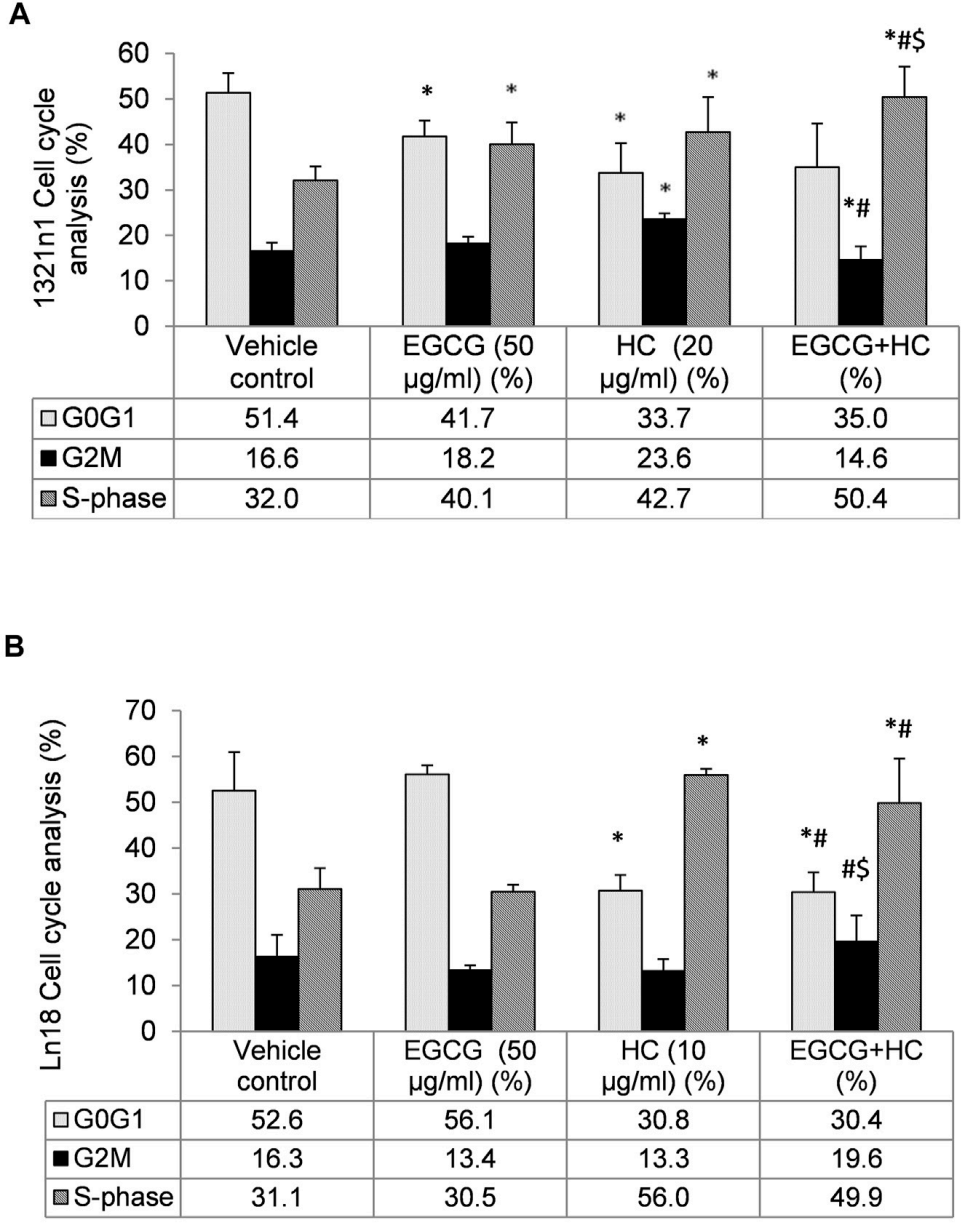
The distribution of **(A)** 1321N1 and **(B)** LN18 cells in the cell cycle phases. The data indicate the mean±SD (*n* = 3). *p < 0.05 compare to vehicle control, #*p* < 0.05 compare to EGCG, $*p* < 0.05 compare to HC.

The proportion of cells in G0/G1 phase reduced (35% for 1321N1 and 30.4% for LN18, respectively) in EGCG+HC treated cells when compared to vehicle control, while S phase was enhanced in 1321N1 (50.4%) and LN18 (49.9%) compared to the vehicle control and EGCG alone (Figure 6). The G2M phase was marginally reduced (14.6%) compared to the vehicle control (Figure 6A).

### 3.6 The Effect of EGCG+HC on the Migration and Invasion of Glioma Cells

**FIGURE 7:**
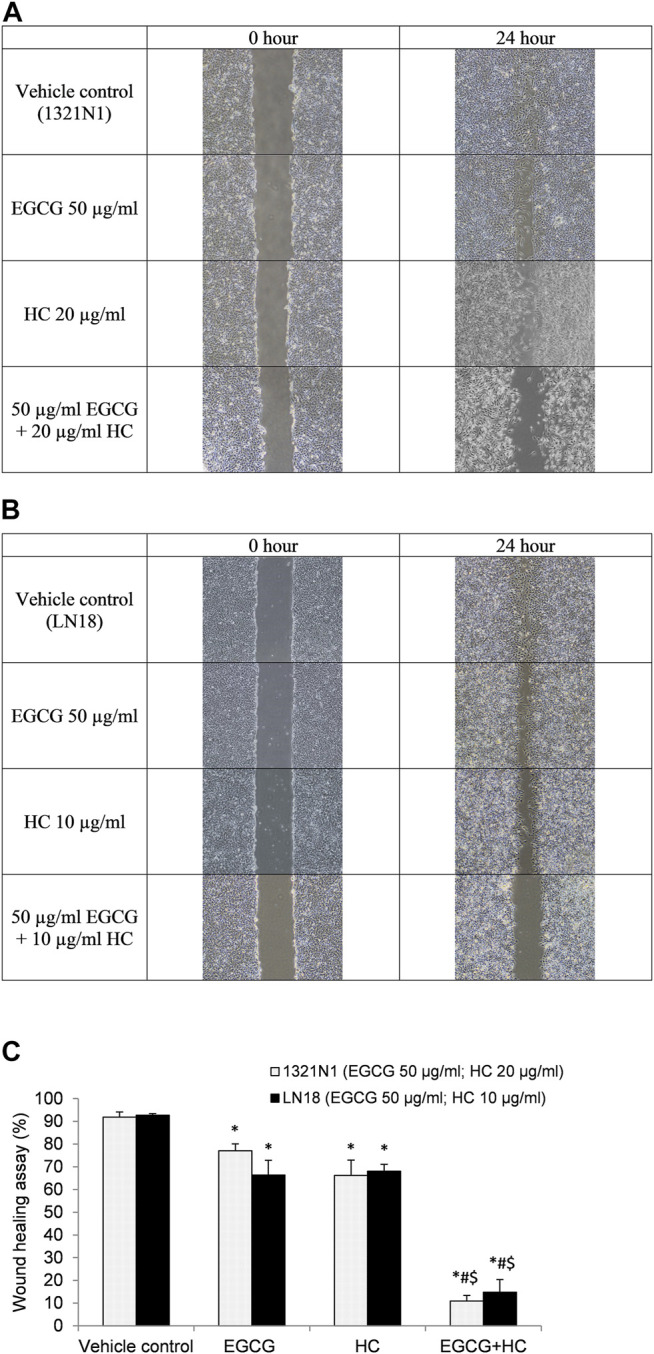
Wound healing assay. The photo was taken at 0- and 24-h following the scratch test. **(A)** 1321N1 cells were treated for 24-h 50 μg/ml EGCG, 20 μg/ml HC, or EGCG+HC, and **(B)** LN18 cells were treated for 24-h with 50 μg/ml EGCG, 10 μg/ml HC, or EGCG+HC. **(C)** The percentage of wound healing closure (%) of 1321N1 and LN18 treated with EGCG, HC, and EGCG+HC. *p < 0.05 compare to control, #*p* < 0.05 compare to EGCG, $*p* < 0.05 compare to HC.

**FIGURE 8:**
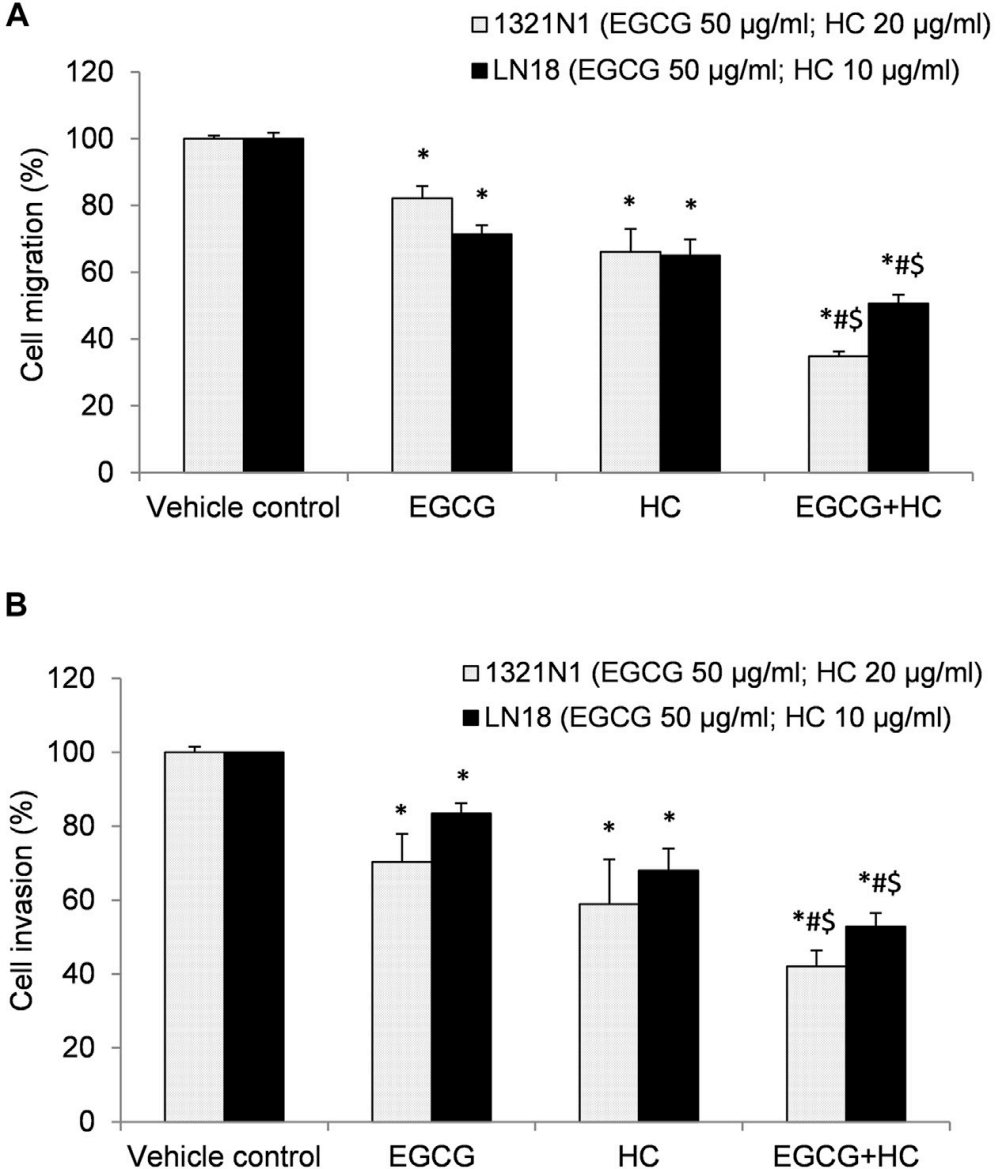
**(A)** Cell migration, and **(B)** cell invasion of 1321N1 and LN18 treated with EGCG+HC. The proportion of cell migration/invasion was represented as a percentage of the vehicle control. Each bar reflects the mean ± SD determined from three independent experiments. *p < 0.05 compare to control, #*p* < 0.05 compare to EGCG, $*p* < 0.05 compare to HC.

In the wound healing experiment, 1321N1 and LN18 cells treated with EGCG+HC had lower migratory potential, with only 10.9 and 14.8% of cells migrated, respectively, as compared to the vehicle control and cells treated with EGCG/HC alone (Figure 7). Transwell migration assays revealed a similar outcome, with less 1321N1 (34.8%) and LN18 (50.7%) EGCG+HC treated cells migrated across the membranes compared to the vehicle control and EGCG/HC alone (Figure 8A). A thin coating of ECM was utilised as an impediment to non-invasive cells *in vitro* in the transwell invasion experiment. As indicated in Figure 8B, EGCG+HC treated cells had lower percentage of 1321N1 and LN18 cell invasion (42.1 and 52.8% respectively) compared to vehicle control and EGCG/HC alone.

### 3.7 The Effect of EGCG+HC on Colony Formation of Glioma Cells

**FIGURE 9:**
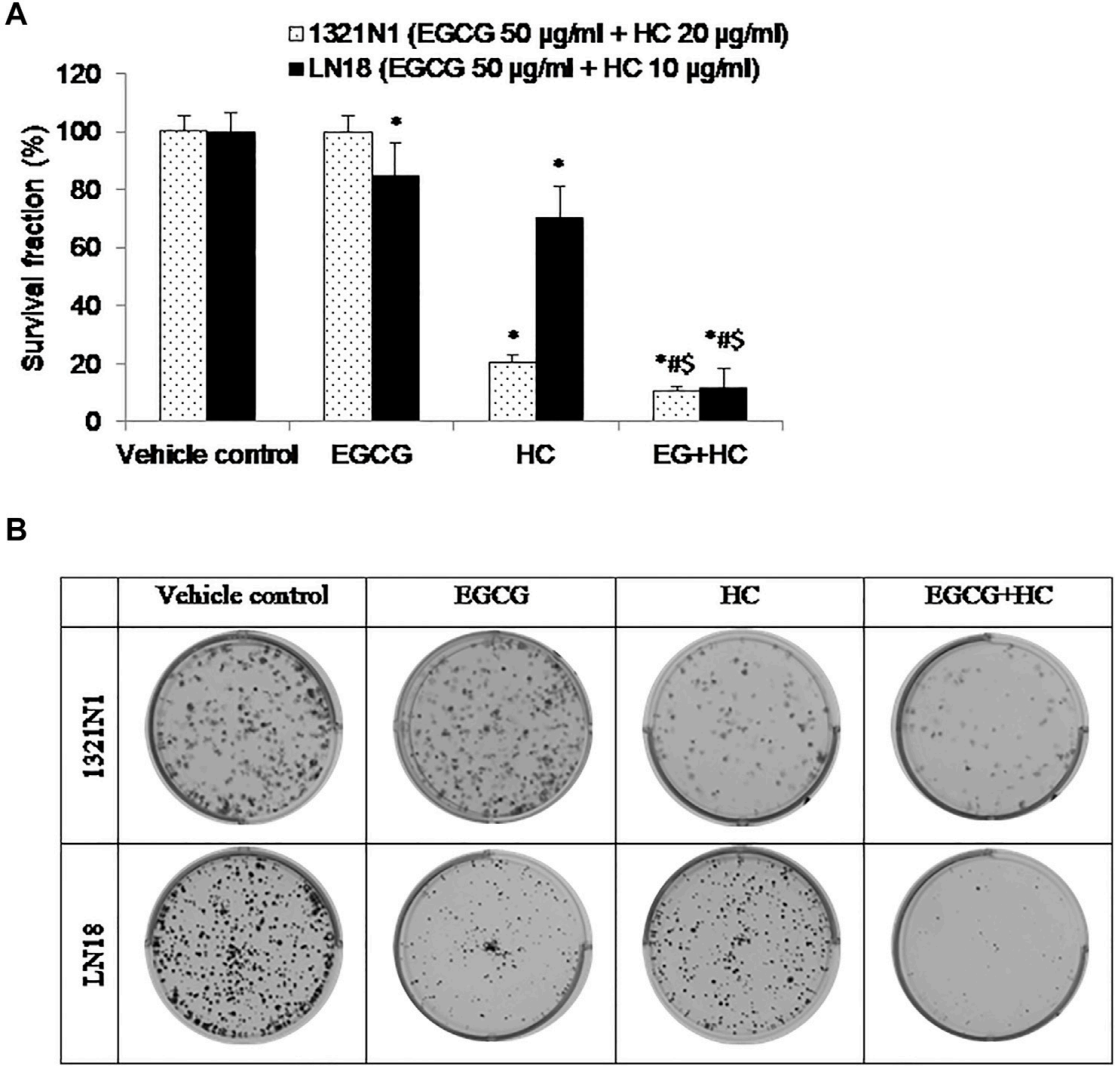
**(A)** The bar graph depicts the suppression of glioma cell colony formation by EGCG, HC, and EGCG+HC in 1321N1 and LN18 cells. **(B)** 1321N1 and LN18 cell colonies treated with EGCG, HC, and EGCG+HC. Each bar reflects the mean±SD determined from three independent experiments. *p < 0.05 compare to control, #*p* < 0.05 compare to EGCG, $*p* < 0.05 compare to HC.

EGCG+HC treatment was more effective than vehicle control and EGCG/HC singly in preventing 1321N1 and LN18 cell colony formation with 10.5 and 11.8% of colonies survived, respectively (Figure 9).

### 3.8 Glioma Cell Lines Expression Profiles

Supplementary Table S1 illustrates a total of 2103 and 2442 differentially expressed genes (FDR *p* ≤ 0.05 and fold change (FC) ≥ 1.5) in 1321N1 and LN18, respectively, treated with EGCG+HC compared to controls. The list of the most significantly expressed genes with the highest FC is shown in Supplementary Tables S2A,B. Approximately 52.8% (1321N1) and 61.5% (LN18) genes were downregulated in EGCG+HC treated cells compared to controls. According to the hierarchical clustering analysis, all of the control and treatment groups were well separated and grouped based on their expression similarity (Supplementary Figure S2). The red colour represented elevated genes, whereas the green colour represented downregulated genes.

### 3.9 Glioma Cell Lines Transcript and Alternative Splicing Expression Changes

Approximately 3782 (1321N1) and 4793 (LN18) transcripts were differently expressed in EGCG+HC treated cells when compared to controls (FDR *p* ≤ 0.05 with fold change (FC) ≥ 1.5) (Supplementary Table S3). The list of the most significantly expressed genes with the highest FC is shown in Supplementary Tables S4A,B. The results revealed that around 27% of transcripts in both cell lines were selectively expressed, with no alterations seen in genes corresponding to the transcripts expressed (Supplementary Table S5). The Partek^®^ Genomics Suite and Tuxedo were used to evaluate the transcripts implicated in alternative splicing events (Supplementary Table S6). The alternative splicing expression generated from Partek ∩ Tuxedo ∩ transcript (FC ≥ 1.5) is provided in the Supplementary Tables S7A,B. Alternative splicing expression of DYNLL1 (downregulated) and DDX39B (upregulated) transcript were altered in 1321N1, whereas RBMX (downregulated) and SEC31A (downregulated) transcript were altered in LN18 treated with EGCG+HC.

### 3.10 Pathway Analysis and Functional Enrichment

Table 3 (GSEA)summarised the biological pathways and the number of genes implicated (control vs treatment). The most significant biological pathway that is upregulated in EGCG+HC treated cells is the endoplasmic reticulum (ER) unfolded protein response (UPR), followed by the activation of the inflammatory response pathway. EGCG+HC downregulated pathways such as mitotic cell cycle control, telomere maintenance, and DNA repair. FET analysis (gene dataset (*p < 0.05, FDR)*) reveals that the apoptotic process, axon guidance and cell cycle arrest were the most substantially enriched BP in EGCG+HC treated groups (Table 4). The selective effects of EGCG+HC on various gene targets in each cell line are most likely responsible for the differences in cellular response seen between the glioma cell lines. Subnetwork studies of gene lists, for example, revealed that major regulator genes such as *MYC, TGFB1, EGR1,* and *KLF4* were present in 1321N1 cell lines treated with EGCG+HC *(p < 0.05, FDR)*. *E2F4, MTOR, E2F1*, and *BRCA* were among the LN18-specific central regulator genes (Table 5).

**TABLE 3:** Gene set enrichment analysis (GSEA). Positive median changes imply increased biological process regulation, whereas negative median changes indicate decreased biological process regulation in cells treated with EGCG+HC vs vehicle control cells.

Gene Significant Enrichment Analysis (GSEA)	1321N1 (no. entities)	1321N1 Median Change	1321N1 *p-*value	LN18 (no. entities)	LN18 Median Change	LN18 *p*-value
endoplasmic reticulum unfolded protein response	80	125.41	0.00E+00	81	127.65	0.00E+00
inflammatory response	253	104.68	9.00E-04	n/a	n/a	n/a
activation of signaling protein activity involved in unfolded protein response	62	103.14	5.00E-04	63	105.67	0.00E+00
immune response	252	102.46	1.40E-03	n/a	n/a	n/a
intrinsic apoptotic signaling pathway in response to endoplasmic reticulum stress	25	102.11	6.00E-04	25	65.48	1.20E-03
cellular metabolic process	132	−47.04	0.00E+00	843	−123.598	0
axon guidance	316	−28.946	0	320	−77.104	0
mitotic cell cycle	387	−108.81	0.00E+00	388	−225.07	0.00E+00
telomere maintenance	54	−53.25	0.00E+00	54	−94.39	0.00E+00
cell division/cytokinesis	88	−51.06	0.00E+00	52	−86.52	0.00E+00
DNA repair	270	−49.21	0.00E+00	270	−154.39	0.00E+00

**TABLE 4:** Fisher exact test (FET) analysis on gene dataset of *p* < 0.05, FDR.

Biological process/signalling pathway	1321N1 No. of overlapping entities	1321N1 *p-*value	LN18 o. of overlapping entities	LN18 *p*-value
positive regulation of apoptotic process/apoptotic process	50	2.95E-10	137	5.68902E-14
axon guidance	57	3.20E-10	60	4.47362E-08
cell cycle arrest	29	1.31E-07	33	1.07385E-07
cellular protein metabolic process	78	4.30E-16	119	2.15597E-33
endoplasmic reticulum unfolded protein response	23	8.59E-09	32	1.24068E-13
mitotic cell cycle	91	4.1441E-31	128	7.19555E-52
mRNA metabolic process	48	5.29E-12	90	5.3289E-37
mRNA splicing, via spliceosome	47	5.10E-16	64	1.57876E-24
negative regulation of cell growth	34	7.11E-11	22	0.003878426
nuclear-transcribed mRNA catabolic process, nonsense-mediated decay	26	4.4963E-07	58	7.18064E-29
response to DNA damage stimulus	46	8.6222E-05	78	6.22536E-14
response to drug	71	1.36E-08	76	1.41321E-06
small molecule metabolic process	155	1.46E-11	202	2.82555E-17
SRP-dependent co-translational protein targeting to membrane	20	6.1778E-05	58	4.58081E-33
Actin Cytoskeleton Regulation	86	2.78E-03	100	0.013074903
Focal Adhesion Regulation	50	2.94E-02	n/a	n/a
Hedgehog Pathway	92	1.24E-02	120	0.001067468
Insulin Action	n/a	n/a	199	2.90484E-10
Notch Pathway	n/a	n/a	248	0.010106517

**TABLE 5:** Subnetwork enrichment analysis on gene dataset of *p* < 0.05, FDR.

Expression target	1321N1 No. of overlapping entities	1321N1 *p-*value	LN18 No. of overlapping entities	LN18 *p*-value
MYC	113	2.93E-17	n/a	n/a
AKT1	137	2.35E-16	136	5.16E-10
TGFB1	228	3.31E-13	n/a	n/a
HIF1A	117	1.98E-12	117	1.28E-07
EGR1	77	4.94E-12	n/a	n/a
JUN	105	5.20E-12	n/a	n/a
NFE2L2	72	7.90E-12	75	2.05E-09
KLF4	53	9.72E-11	n/a	n/a
MAP2K1	54	1.95E-10	n/a	n/a
FGF2	107	3.23E-09	n/a	n/a
ATF4	37	3.36E-09	37	3.96E-07
PARP1	41	4.13E-09	n/a	n/a
FOXM1	46	2.05E-08	n/a	n/a
EIF2AK3	27	2.60E-08	25	1.19E-05
NFYA	26	3.20E-08	n/a	n/a
EDN1	60	4.08E-08	n/a	n/a
ATF3	32	4.44E-08	n/a	n/a
VEGFA	76	6.04E-08	n/a	n/a
E2F4	n/a	n/a	27	1.04E-08
MTOR	n/a	n/a	88	1.24E-08
E2F1	n/a	n/a	74	5.72E-08
BRCA1	n/a	n/a	34	8.09E-08
SRSF3	n/a	n/a	13	6.18E-07
HSP90AA1	n/a	n/a	38	3.00E-06
CUL4A	n/a	n/a	11	8.03E-06
VCP	n/a	n/a	13	1.66E-05
CAPN2	n/a	n/a	16	2.17E-05
CDC20	n/a	n/a	12	2.93E-05
CUL1	n/a	n/a	12	2.93E-05
EGFR	n/a	n/a	70	3.17E-05
DDIT3	n/a	n/a	28	3.68E-05
NRG1	n/a	n/a	40	4.68E-05
SOD1	n/a	n/a	20	6.78E-05
SREBF1	n/a	n/a	41	6.83E-05

### 3.11 RNA-Seq Data Validation

**FIGURE 10:**
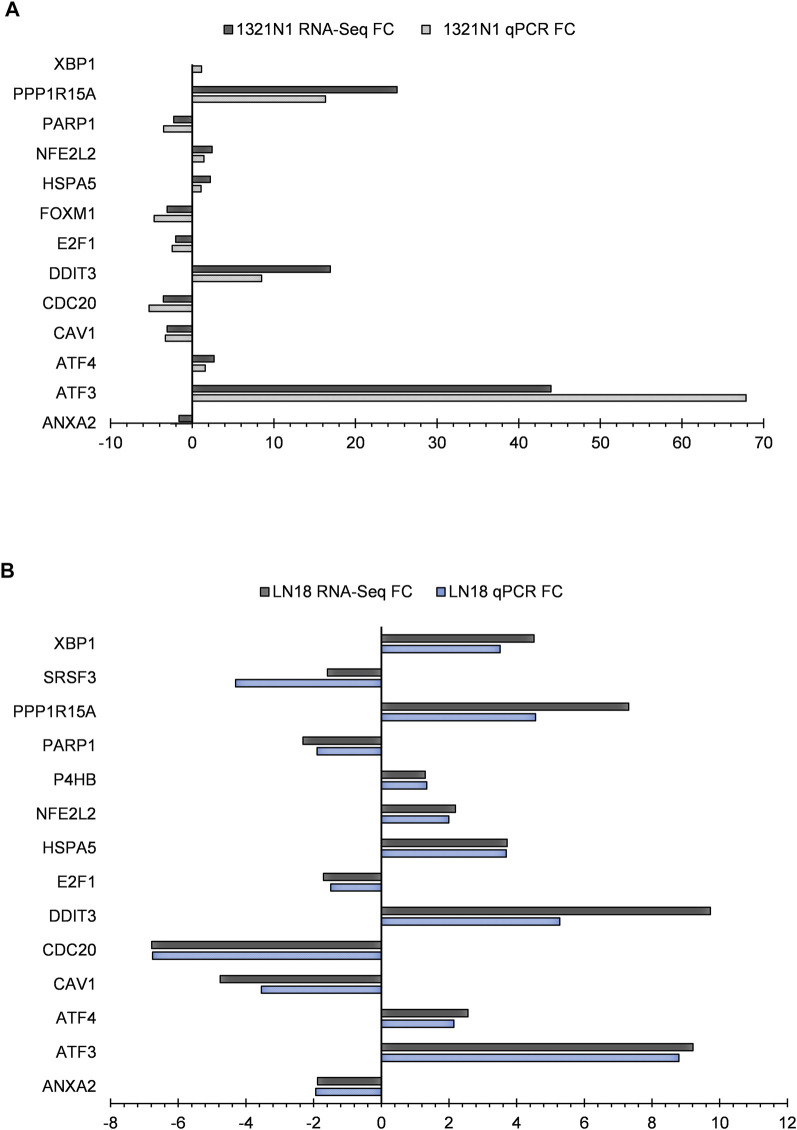
RNA-seq gene expression and genes confirmed by qPCR in EGCG+HC treated cells: **(A)** 1321N1 and **(B)** LN18.

qPCR was used to validate 14 genes from lists of biological processes provided by GSEA, FET, and analysis of gene regulatory subnetworks, as well as three transcripts implicated in alternative splicing events in LN18. Additionally, 13 genes were validated which included three transcripts implicated in alternative splicing events in 1321N1. All the genes and conditions tested were parallel with RNA-seq results (Figure 10).

## 4 Discussion

The purpose of combination therapy is to achieve a synergistic therapeutic effect using lower doses to lessen the toxicity of each agent, and to slow down the induction of drug resistance (Chou, 2010; Zhao et al., 2020) since multiple signal pathways are targeted during the treatment. Previous research found that EGCG increased the efficacy of temozolomide and metformin in U87MG cells and rat C6 glioma cell lines, indicating that EGCG might be a useful adjuvant for cancer chemoprevention (Kuduvalli et al., 2021). Recent literature suggests that HC synergizes with buthionine sulfoximine (BSO), a glutathione synthesis inhibitor to eliminate chronic myeloid leukemic (CML) cells through the GSH-ROS-JNK-ERK-iNOS mediated pathway (Chowdhury et al., 2013) and reports have suggested that HC may be developed as a single-agent chemotherapeutic drug or as an adjuvant (Gundala et al., 2014). However, the effects of EGCG or HC on different stages of glioma cancer cell growth inhibition have not been compared. Furthermore, there has been little research on the interactions of these bioactives, as well as their mode of action on glioma cell death and other inhibitory pathways.

While the concentration of EGCG+HC utilised for 1321N1 and LN18 cells was not harmful to normal WRL68 and normal foreskin fibroblast cells, normal cell proliferation was significantly reduced when treated with the combination dose of SW1783 (100 μg/ml EGCG + 25 μg/ml HC). Different responses to EGCG+HC treatment suggested that the mechanisms by which the combined EGCG+HC act differ in 1321N1, SW1783, and LN18 due to each cell line’s unique mutation. The grade II 1321N1 and grade III SW1783 cell lines both had mtDNA mutations in the coding region, which controls the expression of respiratory complex genes (Soon et al., 2017). They were shown to have decreased mitochondrial activity, and 1321N1 cells were found to have high oxidative stress level. Interestingly, Grade IV LN18 cells do not have any non-synonymous mtDNA mutations and possess high antioxidant capability (Soon et al., 2017). As cancer cells have higher ROS concentrations than normal cells, a high polyphenol concentration is required to enhance the baseline level ROS formation and tilt the redox balance in cancer cells to induce cell death (Harris and Denicola, 2020). The combined dose of 100 μg/ml EGCG + 25 μg/ml HC for SW1783 may have elevated ROS generation over the baseline level of normal cells, disrupting the homeostatic balance of ROS and ultimately resulting in cytotoxicity in normal cells.

Phenolic compounds with pyrogallol groups (EGCG) and/or catechol (HC) are known for their antioxidative effects (Almatroodi et al., 2020; Zamakshshari et al., 2021) as well as their pro-oxidative properties (Gundala et al., 2014; Eghbaliferiz and Iranshahi, 2016; Chen et al., 2020). The utility of antioxidants as an adjuvant with conventional chemotherapy in cancer patients is debatable (Saeidnia and Abdollahi, 2013), due to research indicating that antioxidants may protect cancer cells and impair the efficiency of cytotoxic treatment (Khurana et al., 2018). For example, excessive dosages of beta carotene or vitamin E activity can hasten the progression of lung cancer in smokers (Middha et al., 2019). In contrast, vitamin C and E supplementation may be beneficial in preventing against chemotherapy-related adverse effects (Suhail et al., 2012; Roa et al., 2020). On the other hand, besides their dual roles in scavenging and/or utilizing reactive oxygen species (ROS) to kill cancer cells (Kirtonia et al., 2020), dietary antioxidants possess other anticancer effects as shown by EGCG inhibiting human lymphoma cell proliferation by modulating the epigenetic modification of p16INK4a (Wu et al., 2013), and halting the proliferation of triple-negative breast cancer cells via epigenetic changes of cIAP2 gene (Steed et al., 2020).

In the present study, EGCG+HC treatment inhibited the proliferation of glioma cells, by arresting these cells in the S phase and decreasing the G0/G1 phase to a greater extent than either agent alone. Shen et al. (2014) showed that EGCG (<60 µg/ml) induced apoptosis and cause S phase arrest in hepatocellular carcinoma via the supression of Akt pathway. Similarly, the induction of apoptosis in EGCG treated HT-29 colon cancer cells was reported to involve the p38MAPK activity and Akt pathways (Cerezo-Guisado et al., 2015). Meanwhile, HC was shown to be effective in halting the cell cycle progression of prostate cancer and oral KB carcinoma cells (Gundala et al., 2014). Our results further reveal that EGCG+HC inhibit the migration, invasion, and colony formation of 1321N1 and LN18. Consisent with our findings, EGCG has been shown to suppress A549 lung cancer cell growth and reduce vascular endothelial growth factor (VEGF) expression suggesting its role in the suppression of angiogenesis (Sakamoto et al., 2013). The invasion inhibitory properties of EGCG on thyroid carcinoma 8505C cells was reported via the TGF-β1/Smad signaling through the decrease of epithelial to mesenchymal transition (EMT) markers (Li et al., 2019), while the inhibition of invasion and migration of HeLa, cervical cancer cells were through the modulation of MMP-9 and TIMP-1 (Sharma et al., 2012). Moreover, HC was reported to inhibit the colony formation of prostate cancer cells (Gundala et al., 2014). Limited information is available on the ability of HC to halt the migration/invasion of cancer cells.

Our transcriptomic analysis demonstrated that the molecular mechanism of EGCG+HC against glioma cells is via the down regulation of axon guidance and metabolic pathways. The mechanism of EGCG+HC may be through the downregulation of SEMA3A and SEMA3F transcript expression which may play some roles in inhibiting the glioma proliferation and halts invasion via Plexin A1 (PLXNA1) and B2 (PLXNB2) receptors. Prior research has shown that Semaphorin 3A (SEMA3A), which is known for its axon guidance and antiangiogenic properties, has been implicated in glioblastoma development. Interestingly, SEMA3A was reported to inhibit BTSC proliferation, while inducing invasion where its action is dependent on NRP1 or PLXNA1 receptors. On the other hand, a decrease in SEMA3A receptor expression is enough to stop proliferation and enhance invasion (Higgins et al., 2020). High SEMA2A and PLXNA1 expression are all associated with poorer overall survival in GBM. Similarly, PLXNB2 which was found to be downregulated with EGCG+HC in this study, is recognized a potential biomarker for high-grade glioma. Its knockdown was reported to halt malignant glioma invasion and perivascular diffusion (Le et al., 2015; Huang et al., 2021). Besides the family of semaphorins mentioned above, SEMA7A, downregulated in both 1321N1 and LN18 cells in this study, plays a significant role in mediating the cross-talk between exosomes produced by glioma stem cells (GSC) and the glioma microenvironment (Manini et al., 2019). This further emphasize the axon guidance pathway as an interesting new therapeutic target to curb glioma progression.

Exosomes have been shown to act as signaling mediators of the tumor microenvironment (TME) regulation. Studies indicated that exosomes may transport functional molecules to the recipient cells and aid cancer growth by altering the metabolism of cancer cells and nearby stromal cells (Yang et al., 2020). We postulate that EGCG+HC treatment may inhibit the glioma cancer cells by obstructing the cells’ metabolic reprogramming hence depriving these fast-growing cells of their energy demands. On a similar note, MT-CO3 (down regulated in this study), also involved in the metabolism process specifically the oxidative phosphorylation, influence abnormal energy metabolism and facilitate the growth of tumor cells. Levodopa was shown to inhibit the proliferation of esophageal squamous cell carcinoma (ESCC) via down-regulating the levels of oxidative phosphorylation proteins which includes MT-CO3, SDHD and NDUFS4 (Li et al., 2020). This inhibition is related to mitochondrial dysfunction. Interestingly, miR-5787 was suggested to regulate cisplatin chemoresistance of tongue squamous cell carcinoma (TSCC) by downregulating MT-CO3 which in turn disrupted glucose metabolism (Chen et al., 2019). MT-RNR2, upregulated in both 1321N1 and LN18 cells, is linked to anti-apoptotic activities in bladder cancer (Omar et al., 2017). Although the Warburg theory indicated decreased reliance on mitochondrial function may enhance resistance to apoptosis, studies on the association of mitochondrial genes in cancer progression is limited and this warrants further research (Beadnell et al., 2018).

Genes related to endoplasmic reticulum unfolded protein response (ER UPR) (DDIT3, ATF4, EIF2AK3, XBP1) were mostly found to be upregulated in response to EGCG+HC treatment. UPR plays a vital role in malignant transformation, as well as the regulation of cancer migration and invasion (Limia et al., 2019). Emerging reports have shown the importance of inducing ER stress pathway in cancer treatments, for example, the combination of lopinavir and ritonavir, a protease inhibitor, promotes urological cancer cell death (Okubo et al., 2019). Our previous study has also shown that ER UPR was induced in 1321N1, SW1783 and LN18 cells treated with combined gamma-tocotrienol and hydroxychavicol (Abdul Rahman et al., 2019). Although ER stress seemed to have pertinent role in the anticancer properties of EGCG+HC, further investigation is needed to elucidate whether this induction crosstalk with ROS production or autophagy to unveil the potential regulatory mechanisms of ER-UPR for therapeutic purposes.

Transcriptomic data provides an enormous set of data that can be analysed simultaneously. Our findings are far from exhaustive and may be further explored in terms of the long noncoding genes and alternative splicing expression patterns. For instance, DYNLL1 alternative splicing expression, which is significantly decreased in EGCG+HC treated glioma cells, is reported to be upregulated in gastric cancer high-risk group patients and hepatocellular cancer (Berkel and Cacan, 2020) (Li et al., 2021). How DYNLL1 promotes aberrant transcription in cancers are still unknown.

Despite the fact that only three types of cell lines were examined in this study, the mutations in these cell lines are diverse and reflect different grades of glioma malignancy. Our *in vitro* findings highlighted important points concerning personalised medicine; different dose combinations are required for different grades/mutations, and an increase in grade does not always necessarily require an increase in treatment concentration (50 ug/ml EGCG + 20 ug/ml HC for grade II 1321N1; 50 ug/ml EGCG + 10 ug/ml HC for grade IV LN18). However, insufficient drug exposure was suggested to be one of the contributing factors to the development of resistance to RTK-targeted therapies in glioblastoma due to the heterogeneous expression of the epidermal growth factor receptor (EGFR) (Furnari et al., 2015). Our findings warrant further elucidation on the significance of having specific treatment doses for different glioma grades.

## 5 Conclusion

EGCG+HC can potentiate the S phase arrest and induce the activation of caspase-3 to initiate apoptosis and inhibit the cell proliferation of 1321N1 and LN18 glioma cells. Furthermore, the strong inhibition of migration, invasion and colony formation in EGCG+HC treated cells indicated enhanced efficacy of combined EGCG and HC compared to single treatments. EGCG+HC exerted its apoptotic effect through the alteration of mitochondrial genes and metabolic pathways, while simultaneously interfering with endoplasmic reticulum unfolded protein response and axon guidance pathway. Crosstalk between activated pathways might be a significant regulator of glioma cell response to EGCG+HC treatment, making it a promising therapeutic target. Further research on the possible interactions between metabolic pathway, endoplasmic reticulum unfolded protein response, and axon guidance signalling in glioma is required.

## Data Availability

The RNA-seq dataset presented in this study have been deposited in the NCBI Gene expression omnibus (GEO) database and are accessible through the accession number GSE193838 (https://www.ncbi.nlm.nih.gov/geo/query/acc.cgi?acc=GSE193838).
