# Supplementary material for: Inhibitory Mechanism of Combined Hydroxychavicol With Epigallocatechin-3-Gallate Against Glioma Cancer Cell Lines: A Transcriptomic Analysis
Source: Front Pharmacol. 2022 Mar 22;13:844199. doi: 10.3389/fphar.2022.844199 (PMC8982671; doi:10.3389/fphar.2022.844199)
Supplement: Supplementary file 1 [file Table5.pdf]

Table S5 Comparison between total gene and transcript expressed significantly with *P*-value FDR ≤ 0.05, and fold change (FC) ≥ 1.5.

Regulation of the entities	1321N1 EGCG+HC vs. Control	LN18 EGCG+HC vs. Control
Genes	2103	2442
Transcripts	3782	4793
Present in both of the entities lists (Genes ∩ Transcripts)	1218	1592
Unique to Genes	885	850
Unique to Transcripts	1044	1292
