# Supplementary material for: Inhibitory Mechanism of Combined Hydroxychavicol With Epigallocatechin-3-Gallate Against Glioma Cancer Cell Lines: A Transcriptomic Analysis
Source: Front Pharmacol. 2022 Mar 22;13:844199. doi: 10.3389/fphar.2022.844199 (PMC8982671; doi:10.3389/fphar.2022.844199)
Supplement: Supplementary file 2 [file Table4.pdf]

Table S4 A. Lists of 20 transcripts with the lowest *P*-value, and FDR from *t*-test statistical analysis for 1321N1 cells treated with EGCG+HC.

Transcript ID	Gene name	Ensembl ID	Fold change 3EGCG+HC	*P*-value with FDR 3EGCG+HC	Fold change 3EGCG	*P*-value with FDR 3EGCG	Fold change 3HC	*P*-value with FDR 3HC
HMOX1_4	HMOX1	ENST00000216117	140.81	0.00E+00	n/a	n/a	105.26	0.00E+00
HSPA1B_2	HSPA1B	ENST00000375650	32.38	3.04E-128	1.49	2.70E-02	80.71	0.00E+00
RTN4_10	RTN4	ENST00000394609	20.24	1.32E-121	n/a	n/a	n/a	n/a
RND3_5	RND3	ENST00000263895	12.33	1.46E-115	n/a	n/a	10.72	1.56E-109
RPL27A_6	RPL27A	ENST00000534599	11.20	1.32E-121	n/a	n/a	3.42	3.12E-08
BAG3_1	BAG3	ENST00000369085	7.84	2.37E-131	n/a	n/a	8.10	1.47E-142
HSP90AA1_10	HSP90AA1	ENST00000557089	6.80	7.56E-147	n/a	n/a	3.69	2.31E-12
SQSTM1_17	SQSTM1	ENST00000466342	6.60	1.40E-126	1.99	2.83E-03	10.58	7.96E-134
DNAJB1_5	DNAJB1	ENST00000254322	5.94	2.31E-119	n/a	n/a	14.61	7.14E-75
PTP4A1_3	PTP4A1	ENST00000370651	5.64	5.89E-152	n/a	n/a	2.69	2.83E-24
ACTB_10	ACTB	ENST00000462494	-4.97	0.00E+00	-1.49	3.40E-02	-4.45	0.00E+00
TUBB_3	TUBB	ENST00000327892	-2.71	0.00E+00	-1.18	4.30E-02	-4.07	0.00E+00
TMSB4X_4	TMSB4X	ENST00000451311	-2.66	0.00E+00	n/a	n/a	-2.55	1.74E-13
MT-CO3_1	MT-CO3	ENST00000362079	-2.43	0.00E+00	n/a	n/a	-2.17	0.00E+00
ACTB_4	ACTB	ENST00000464611	-2.35	0.00E+00	-1.26	4.95E-11	-3.55	0.00E+00
MT-ND2_1	MT-ND2	ENST00000361453	-2.29	0.00E+00	-1.16	1.90E-02	-1.97	0.00E+00
PTMA_8	PTMA	ENST00000341369	-2.24	0.00E+00	n/a	n/a	-2.45	0.00E+00
MT-ATP6_1	MT-ATP6	ENST00000361899	-2.21	0.00E+00	n/a	n/a	-1.89	0.00E+00
MT-ND3_1	MT-ND3	ENST00000361227	-2.20	0.00E+00	-1.23	3.20E-02	-2.01	0.00E+00
MT-CYB_1	MT-CYB	ENST00000361789	-2.14	0.00E+00	n/a	n/a	-1.82	0.00E+00

Table S9 B. Lists of 20 transcripts with the lowest *P*-value, and FDR from *t*-test statistical analysis for LN18 cells treated with EGCG+HC.

Transcript ID	Gene name	Ensembl ID	Fold change LNEGCG+HC	*P*-value with FDR LNEGCG+HC	Fold change LNEGCG	*P*-value with FDR LNEGCG	Fold change LNHC	*P*-value with FDR LNHC
SLC3A2_5	SLC3A2	ENST00000539458	27.85	4.76E-115	2.55	3.76E-14	2.27	9.99E-10
RPS8_2	RPS8	ENST00000485390	21.66	2.79E-121	n/a	n/a	n/a	n/a
RND3_2	RND3	ENST00000375734	15.14	3.14E-134	2.10	7.44E-03	n/a	n/a
RPL35_5	RPL35	ENST00000487431	9.14	1.94E-127	n/a	n/a	n/a	n/a
SQSTM1_17	SQSTM1	ENST00000466342	8.74	4.33E-119	3.34	1.11E-102	2.04	1.62E-04
RPL27A_6	RPL27A	ENST00000534599	5.54	2.49E-134	2.63	3.73E-09	n/a	n/a
RPS13_1	RPS13	ENST00000525828	5.49	1.16E-127	2.11	5.65E-03	n/a	n/a
SLC3A2_22	SLC3A2	ENST00000539891	4.99	7.98E-122	5.61	7.84E-16	n/a	n/a
CANX_20	CANX	ENST00000508787	4.55	3.46E-139	3.08	7.98E-10	n/a	n/a
HMOX1_4	HMOX1	ENST00000216117	3.90	1.92E-127	2.17	2.40E-02	2.93	5.79E-06
F3_2	F3	ENST00000334047	-22.62	1.81E-148	-3.24	2.02E-44	n/a	n/a
DIO2_4	DIO2	ENST00000555750	-12.69	7.38E-76	n/a	n/a	-3.77	3.02E-29
PRSS23_3	PRSS23	ENST00000280258	-6.62	1.11E-100	n/a	n/a	-2.15	8.84E-20
CAV1_6	CAV1	ENST00000393467	-5.87	3.43E-112	-1.52	6.33E-07	-1.51	7.26E-08
MT2A_2	MT2A	ENST00000245185	-3.68	4.87E-89	-6.06	2.46E-48	n/a	n/a
HSP90AA1_7	HSP90AA1	ENST00000216281	-2.55	0.00E+00	n/a	n/a	-1.42	1.38E-06
ACTG1_4	ACTG1	ENST00000331925	-2.34	0.00E+00	n/a	n/a	-1.75	3.54E-08
TUBB_3	TUBB	ENST00000327892	-2.31	0.00E+00	-1.21	6.59E-03	-1.76	0.00E+00
EIF4A1_8	EIF4A1	ENST00000584784	-1.96	0.00E+00	-1.29	3.80E-02	-2.02	6.05E-12
TUBA1B_1	TUBA1B	ENST00000550367	-1.58	0.00E+00	-1.30	6.34E-10	-1.72	0.00E+00
