# Supplementary material for: Inhibitory Mechanism of Combined Hydroxychavicol With Epigallocatechin-3-Gallate Against Glioma Cancer Cell Lines: A Transcriptomic Analysis
Source: Front Pharmacol. 2022 Mar 22;13:844199. doi: 10.3389/fphar.2022.844199 (PMC8982671; doi:10.3389/fphar.2022.844199)
Supplement: Supplementary file 3 [file Table1.pdf]

Table S1. Total genes expressed at fold change (FC) ≥ 1.5, false discovery rate (FDR) $P \leq 0.05$.

Gene regulation	1321N1 EGCG+HC vs. Control	LN18 EGCG+HC vs. Control
Increased	992	940
Decreased	1111	1502
Total genes	2103	2442
