# Supplementary material for: Inhibitory Mechanism of Combined Hydroxychavicol With Epigallocatechin-3-Gallate Against Glioma Cancer Cell Lines: A Transcriptomic Analysis
Source: Front Pharmacol. 2022 Mar 22;13:844199. doi: 10.3389/fphar.2022.844199 (PMC8982671; doi:10.3389/fphar.2022.844199)
Supplement: Supplementary file 4 [file Table2.pdf]

Table S2 A. Lists of 20 genes with the lowest *P*-value, and FDR from *t*-test statistical analysis for 1321N1 cells treated with EGCG+HC.

Gene	Fold change 3EGCG+HC	*P*-value with FDR 3EGCG+HC	Fold change 3EGCG	*P*-value with FDR 3EGCG	Fold change 3HC	*P*-value with FDR 3HC
RP11-25K21.6	1296.29	0.00E+00	n/a	n/a	3202.88	0.00E+00
HMOX1	117.04	0.00E+00	n/a	n/a	99.11	0.00E+00
HSPA1A	27.33	3.76E-108	n/a	n/a	116.76	2.08E-256
HSPA1B	27.21	0.00E+00	1.41	2.40E-02	77.23	0.00E+00
RND3	9.70	1.49E-114	n/a	n/a	8.16	3.86E-92
CTD-2021J15.1	8.64	1.25E-131	n/a	n/a	n/a	n/a
SLC3A2	7.23	2.92E-126	n/a	n/a	9.67	4.01E-144
BAG3	6.70	2.99E-114	n/a	n/a	7.81	4.98E-139
AC092755.4	5.81	1.38E-123	n/a	n/a	3.28	2.49E-20
PTP4A1	4.75	1.30E-117	n/a	n/a	2.58	1.30E-20
ACTB	-3.51	0.00E+00	-1.52	0.00E+00	-4.02	0.00E+00
MT-CO3	-2.89	0.00E+00	-1.24	6.93E-03	-2.26	0.00E+00
MT-ND2	-2.79	0.00E+00	-1.25	2.30E-04	-2.11	0.00E+00
TUBB	-2.77	0.00E+00	-1.22	4.51E-09	-3.41	0.00E+00
MT-ATP6	-2.67	0.00E+00	n/a	n/a	-2.00	0.00E+00
MT-CYB	-2.56	0.00E+00	-1.27	8.37E-04	-1.91	0.00E+00
MT-ND5	-2.47	0.00E+00	-1.21	1.70E-02	-1.62	0.00E+00
MT-ATP8	-2.44	0.00E+00	n/a	n/a	-1.87	0.00E+00
MT-ND6	-2.27	0.00E+00	n/a	n/a	n/a	n/a
MT-ND1	-2.06	0.00E+00	-1.37	2.60E-07	-1.73	0.00E+00

Table S2 B. Lists of 20 genes with the lowest *P*-value, and FDR from *t*-test statistical analysis for LN18 cells treated with EGCG+HC.

Gene	Fold change LNEGCG+HC	*P*-value with FDR LNEGCG+HC	Fold change LNEGCG	*P*-value with FDR LNEGCG	Fold change LNHC	*P*-value with FDR LNHC
HMOX1	22.87	9.99E-167	n/a	n/a	2.84	1.07E-04
RP11-973N13.4	20.63	1.26E-88	n/a	n/a	n/a	n/a
RP11-1035H13.3	13.69	1.14E-84	2.61	5.49E-03	n/a	n/a
RND3	10.71	1.44E-141	2.37	1.75E-07	1.81	5.29E-03
MT-RNR2	10.21	1.77E-146	2.10	5.65E-70	1.68	3.15E-25
PPP1R15A	7.31	2.12E-114	n/a	n/a	n/a	n/a
AC005027.3	6.08	1.84E-106	1.98	6.83E-04	n/a	n/a
TAF13	4.66	2.19E-80	1.75	3.37E-03	n/a	n/a
RP11-739N20.2	3.11	4.64E-86	n/a	n/a	1.29	3.20E-02
FTL	1.63	1.82E-81	n/a	n/a	n/a	n/a
NEAT1	-6.73	7.85E-149	-2.98	3.29E-50	n/a	n/a
FST	-4.99	3.13E-100	-1.74	1.73E-13	-2.71	1.71E-34
MT2A	-4.30	2.06E-124	-6.08	6.11E-136	n/a	n/a
MYH9	-4.02	1.53E-100	n/a	n/a	-1.44	2.54E-06
ACTB	-2.86	0.00E+00	-1.21	7.53E-03	-2.13	0.00E+00
TUBA1B	-1.86	0.00E+00	-1.35	0.00E+00	-1.74	0.00E+00
TUBB	-1.80	0.00E+00	-1.27	0.00E+00	-1.64	0.00E+00
HSPA8	-1.71	0.00E+00	1.31	3.22E-16	-1.71	0.00E+00
UBB	-1.54	0.00E+00	-1.27	4.86E-09	-1.32	1.64E-05
TPI1	-1.53	0.00E+00	n/a	n/a	n/a	n/a
