# Supplementary material for: Inhibitory Mechanism of Combined Hydroxychavicol With Epigallocatechin-3-Gallate Against Glioma Cancer Cell Lines: A Transcriptomic Analysis
Source: Front Pharmacol. 2022 Mar 22;13:844199. doi: 10.3389/fphar.2022.844199 (PMC8982671; doi:10.3389/fphar.2022.844199)
Supplement: Supplementary file 5 [file Table6.pdf]

Table S6 Comparison between Partek® Genomics Suite analysis and Tuxedo Suite analysis for alternative splicing events (*P*-value FDR ≤ 0.05).

Alternative splicing events	1321N1 EGCG+HC vs. Control	LN18 EGCG+HC vs. Control
Partek	3897	4260
Tuxedo	432	739
Present in both analysis (Partek ∩ Tuxedo)	201	366
Partek ∩ Tuxedo ∩ Transcripts (FC ≥ 1.5)	18	46
