# Supplementary material for: Inhibitory Mechanism of Combined Hydroxychavicol With Epigallocatechin-3-Gallate Against Glioma Cancer Cell Lines: A Transcriptomic Analysis
Source: Front Pharmacol. 2022 Mar 22;13:844199. doi: 10.3389/fphar.2022.844199 (PMC8982671; doi:10.3389/fphar.2022.844199)
Supplement: Supplementary file 6 [file Image2.pdf]

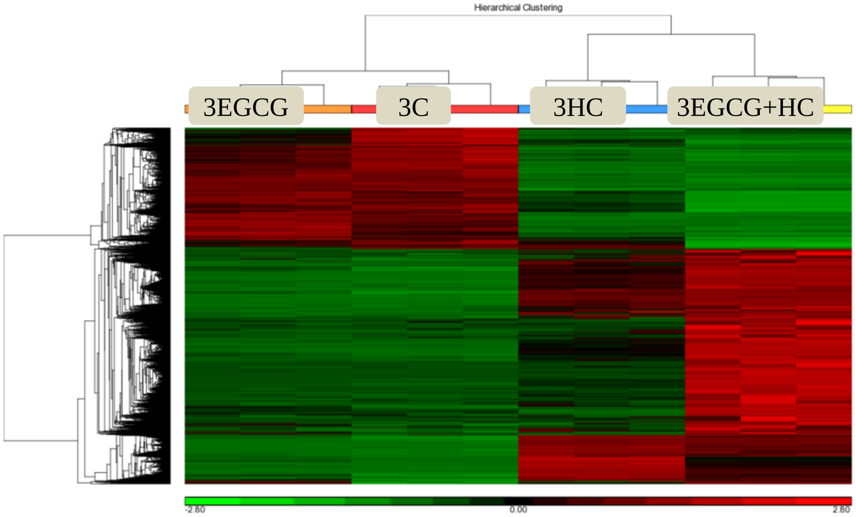

a.

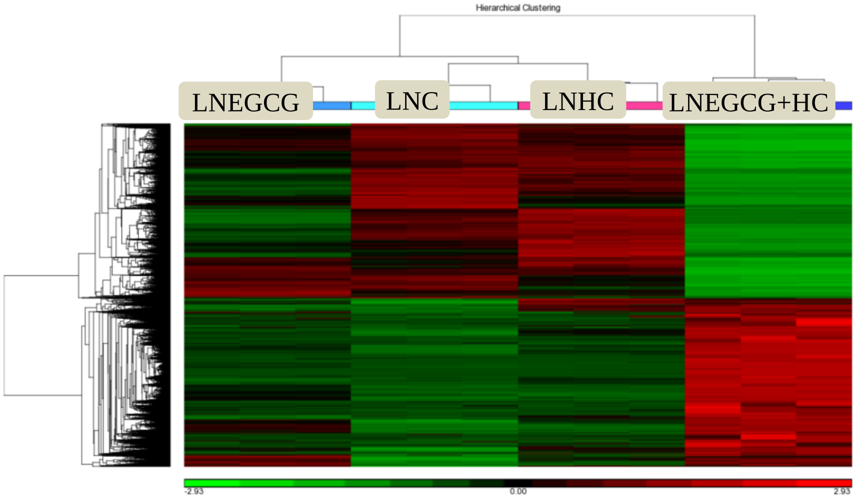

b.

Figure S2 Hierarchical clustering analysis with differential metric distance and centroid network rules for a. 1321N1 and b. LN18 cells treated with EGCG+HC.
