# Supplementary material for: Inhibitory Mechanism of Combined Hydroxychavicol With Epigallocatechin-3-Gallate Against Glioma Cancer Cell Lines: A Transcriptomic Analysis
Source: Front Pharmacol. 2022 Mar 22;13:844199. doi: 10.3389/fphar.2022.844199 (PMC8982671; doi:10.3389/fphar.2022.844199)
Supplement: Supplementary file 7 [file Table3.pdf]

Table S3 Total transcripts expressed at fold change (FC) ≥ 1.5, false discovery rate (FDR) $P \leq 0.05$.

Transcript regulation	1321N1 EGCG+HC vs. Control	LN18 EGCG+HC vs. Control
Increased	2611	2551
Decreased	1171	2242
Total genes	3782	4793
