# Supplementary material for: Inhibitory Mechanism of Combined Hydroxychavicol With Epigallocatechin-3-Gallate Against Glioma Cancer Cell Lines: A Transcriptomic Analysis
Source: Front Pharmacol. 2022 Mar 22;13:844199. doi: 10.3389/fphar.2022.844199 (PMC8982671; doi:10.3389/fphar.2022.844199)
Supplement: Supplementary file 8 [file Table7.pdf]

Table S7 A. Lists of differentially expressed alternative splicing events (FDR $P \leq 0.05$) for Partek ∩ Tuxedo ∩ transcript (FC ≥ 1.5) in 1321N1treated with EGCG+HC.

Number of transcript	Test ID	Gene ID	Gene	Locus	P-value, Alternative splicing events (Partek)	Fold change (3EGCG+HC vs. control) (Partek)	J score (Tuxedo)	P-value (Tuxedo)	q-value (Tuxedo)
7	TSS52585	ENSG00000026508	CD44	11:35160416-35253949	2.02E-20	-1.90	4.75E-01	3.50E-04	3.80E-03
10	TSS144846	ENSG00000068745	IP6K2	3:48725435-48777786	2.96E-19	10.63	7.45E-01	5.00E-05	6.46E-04
5	TSS122886	ENSG00000135535	CD164	6:109687716-109703762	5.49E-12	-2.09	1.72E-01	5.00E-05	6.46E-04
8	TSS192765	ENSG00000168066	SF1	11:64532077-64546258	9.93E-11	2.58	1.04E-01	5.70E-03	4.27E-02
3	TSS36681	ENSG00000075415	SLC25A3	12:98987368-98995946	1.21E-10	-2.79E+05	3.82E-01	4.20E-03	3.38E-02
5	TSS195287	ENSG00000108107	RPL28	19:55896712-55919145	2.44E-10	-229.44	2.47E-01	5.00E-05	6.46E-04
3	TSS89565	ENSG00000160201	U2AF1	21:44513065-44527697	2.10E-08	-197.47	2.69E-01	1.00E-04	1.21E-03
3	TSS24042/ TSS23111	ENSG00000198563	DDX39B	6:31496493-31526606	7.02E-08	25.74	6.39E-01	5.00E-05	6.46E-04
3	TSS6070	ENSG00000088986	DYNLL1	12:120907652-120936296	1.01E-07	-2.53E+10	2.93E-01	5.00E-05	6.46E-04
3	TSS167651	ENSG00000136450	SRSF1	17:56066398-56084707	1.95E-07	-4.59E+03	2.52E-01	5.00E-05	6.46E-04

Table S7 B. Lists of differentially expressed alternative splicing events (FDR $P \leq 0.05$) for Partek ∩ Tuxedo ∩ transcript (FC ≥ 1.5) in LN18 treated with EGCG+HC.

Number of transcript	Test ID	Gene ID	Gene	Locus	P-value, Alternative splicing events (Partek)	Fold change (LNEGCG+HC vs. control) (Partek)	J score (Tuxedo)	P-value (Tuxedo)	q-value (Tuxedo)
10	TSS167866	ENSG00000101150	TPD52L2	20:62496595-62522898	9.38E-29	-5.23	8.99E-02	6.50E-04	4.44E-03
9	TSS187434	ENSG00000182199	SHMT2	12:57623109-57634498	5.47E-25	-37.19	3.45E-01	5.00E-05	4.27E-04
8	TSS103855	ENSG00000197713	RPE	2:210867288-211036115	3.10E-24	-3.88	3.80E-01	4.95E-03	2.53E-02
8	TSS55735	ENSG00000138674	SEC31A	4:83739813-83934079	7.01E-17	10.50	8.17E-01	7.45E-03	3.47E-02
4	TSS192448	ENSG00000091140	DLD	7:107531414-107643700	3.23E-15	-143.85	3.58E-01	5.00E-05	4.27E-04
6	TSS151776	ENSG00000179152	TCAIM	3:44379432-44450943	4.91E-15	2.82	9.28E-02	7.30E-03	3.44E-02
9	TSS111182	ENSG00000140995	DEF8	16:90014332-90034468	5.76E-15	1.98	1.15E-01	1.50E-03	9.29E-03
6	TSS47282	ENSG00000111912	NCOA7	6:126102306-126252266	5.96E-15	8.56	3.86E-01	5.00E-05	4.27E-04
5	TSS108842	ENSG00000049541	RFC2	7:73645828-73668774	1.90E-14	-29.50	1.93E-01	1.05E-02	4.61E-02
4	TSS190329	ENSG00000147274	RBMX	X:135923089-135962923	4.88E-14	-2291.81	3.04E-01	5.00E-05	4.27E-04
