# Supplementary material for: Inhibitory Mechanism of Combined Hydroxychavicol With Epigallocatechin-3-Gallate Against Glioma Cancer Cell Lines: A Transcriptomic Analysis
Source: Front Pharmacol. 2022 Mar 22;13:844199. doi: 10.3389/fphar.2022.844199 (PMC8982671; doi:10.3389/fphar.2022.844199)
Supplement: Supplementary file 9 [file Image1.pdf]

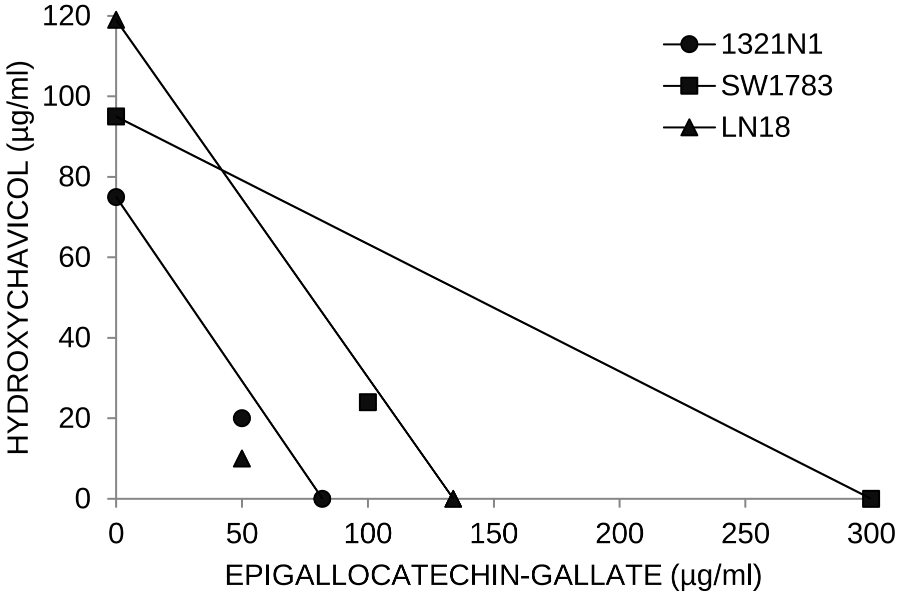

Supplementary Figure S1. Isobologram analysis of the effects of EGCG and HC on glioma cells. The IC50 of EGCG and HC were plotted on the x and y axes. The line connecting these dots depicts the treatment concentrations of each compound that, if administered in combination, would cause the same growth inhibition if the interaction between these compounds was additive. The data point on the isobologram reflects the actual dosages of EGCG+HC that result in a 50% growth inhibition. Since the data point is located significantly below either line, a synergistic anti-proliferative impact is suggested.
